# Supplementary material for: Use of metabolomics for predicting spontaneous preterm birth in asymptomatic pregnant women: protocol for a systematic review and meta-analysis
Source: BMJ Open. 2019 Mar 4;9(3):e026033. doi: 10.1136/bmjopen-2018-026033 (PMC6429842; doi:10.1136/bmjopen-2018-026033)
Supplement: Supplementary data [file bmjopen-2018-026033supp001.pdf]

Seacrh strategy: #1 AND #2 AND #3

1 (OR for each term)	preterm birth<br>premature birth<br>premature infant<br>premature labor<br>extremely premature infant<br>premature obstetric labor<br>spontaneous preterm birth<br>extreme preterm birth<br>late preterm birth<br>moderate preterm birth<br>preterm premature rupture of membranes<br>preterm delivery<br>PROM<br>sPTB<br>preterm PROM<br>pPROM<br>p-PROM
2 (OR for each term)	metabolomic*<br>metabonomic*<br>metabolit*<br>lipidomic*<br>H NMR<br>proton NMR<br>proton nuclear magnetic resonance<br>liquid chromatogra*<br>UPLC<br>ultra-performance liquid chromatograph*<br>ultra performance liquid chromatograph*<br>HPLC<br>high performance liquid chromatograph*<br>high-performance liquid chromatograph*
3 (OR for each term)	pregnan*<br>antenat*<br>ante nat*<br>prenat*<br>pre nat*
