# Supplementary material for: Use of metabolomics for predicting spontaneous preterm birth in asymptomatic pregnant women: protocol for a systematic review and meta-analysis
Source: BMJ Open. 2019 Mar 4;9(3):e026033. doi: 10.1136/bmjopen-2018-026033 (PMC6429842; doi:10.1136/bmjopen-2018-026033)
Supplement: Author's manuscript [file bmjopen-2018-026033.draft_revisions.pdf]

BMJ Open is committed to open peer review. As part of this commitment we make the peer review history of every article we publish publicly available.

When an article is published we post the peer reviewers' comments and the authors' responses online. We also post the versions of the paper that were used during peer review. These are the versions that the peer review comments apply to.

The versions of the paper that follow are the versions that were submitted during the peer review process. They are not the versions of record or the final published versions. They should not be cited or distributed as the published version of this manuscript.

BMJ Open is an open access journal and the full, final, typeset and author-corrected version of record of the manuscript is available on our site with no access controls, subscription charges or pay-per-view fees (http://bmjopen.bmj.com).

If you have any questions on BMJ Open's open peer review process please email editorial.bmjopen@bmj.com

The use of metabolomics for predicting spontaneous preterm birth in asymptomatic pregnant women: protocol for a systematic review and meta-analysis

Journal:	*BMJ Open*
Manuscript ID	bmjopen-2018-026033
Article Type:	Protocol
Date Submitted by the Author:	15-Aug-2018
Complete List of Authors:	Souza, Renato; Universidade Estadual de Campinas, Obstetrics and Gynecology<br>Galvão, Rafael<br>Leite, Debora; Campinas' State University, Department of Tocogynecology; Universidade Federal de Pernambuco,<br>Passini Jr, Renato; Universidade Estadual de Campinas Faculdade de Ciencias Medicas<br>Baker, Philip ; University of Leicester, College of Medicine<br>Cecatti, Jose Guilherme; University of Campinas, Obstetrics and Gynecology
Keywords:	preterm birth, metabolomics, biomarkers, prediction

SCHOLARONE™
Manuscripts

For peer review only

The use of metabolomics for predicting spontaneous preterm birth in asymptomatic pregnant women: protocol for a systematic review and meta-analysis

Renato T. Souza (renatotsouzasp@gmail.com)

Rafael Bessa Freitas Galvão (rafaelbfg@gmail.com)

Débora Farias Batista Leite (deborafariasleite@gmail.com)

Renato Passini Jr (passini@caism.unicamp.br)

Philip N. Baker (philip.baker@leicester.ac.uk)

José Guilherme Cecatti (cecatti@unicamp.br)

Department of Obstetrics and Gynaecology, University of Campinas, Campinas, São Paulo, Brazil.

Clinics Hospital of Federal University of Pernambuco, Recife, Pernambuco, Brazil.

College of Life Sciences, University of Leicester, England, United Kingdom.

This systematic review protocol is registered at PROSPERO platform (CRD42018100172). Available from: http://www.crd.york.ac.uk/PROSPERO/display_record.php?ID=CRD42018100172.

Corresponding author:

Renato T Souza

101 Alexander Fleming, Cidade Universitária, campinas, São Paulo, Brazil.

ZIPCODE 13083-881

renatotsouzasp@gmail.com

Word count: 2,921.

For peer review only - http://bmjopen.bmj.com/site/about/guidelines.xhtml

ABSTRACT

Introduction: Preterm birth (PTB) is the leading cause of neonatal mortality and short- and long-term morbidity. The aetiology and pathophysiology of spontaneous PTB are still unclear, which makes the identification of reliable and accurate predictor markers more difficult, particularly for unscreened or asymptomatic women. Metabolomics biomarkers have been demonstrated to be potentially accurate biomarkers for many disorders with complex mechanisms such as PTB. Therefore, we aim to perform a systematic review of metabolomics markers associated with spontaneous PTB. Our research question is “What is the performance of metabolomics for predicting spontaneous preterm birth in asymptomatic pregnant women?”

Methods and analysis: We will focus on studies assessing metabolomics techniques for predicting spontaneous preterm birth in asymptomatic pregnant women. We will conduct a comprehensive systematic review of the literature from the last 10 years. Only observational cohort and case-control studies will be included. Our search strategy will be carried out by two independent reviewers, who will scan title and abstract before carrying out a full review of the article. The scientific databases to be explored include PubMed, MedLine, ScieLo, EMBASE, LILACS, Web of Science, Scopus, and others.

Ethics and dissemination: This systematic review protocol does not require ethical approval. We intend to disseminate our findings in scientific peer-reviewed journal, the Preterm SAMBA study open access website, specialists’ conferences, and to our funding agencies.

Registration details: This protocol is registered in PROSPERO platform (code CRD42018100172).

Keywords: preterm birth, spontaneous preterm birth, metabolomics, biomarkers, prediction.

For peer review only

For peer review only - http://bmjopen.bmj.com/site/about/guidelines.xhtml

Strengths and limitations of this study

● This systematic review protocol takes into account some important aspects regarding conducting a systematic review about spontaneous preterm birth and metabolomics such as the criteria used for defining spontaneous preterm birth, different population risk stratification, method used to estimate gestational age, and metabolomics techniques details.

● Two independent reviewers are responsible for searching and selecting studies, as also extracting data, and a third reviewer will resolve any disagreement.

● If possible, proper statistical methods will be applied to investigate metabolomics accuracy in predicting spontaneous preterm birth.

For peer review only - http://bmjopen.bmj.com/site/about/guidelines.xhtml

INTRODUCTION

Spontaneous preterm birth (sPTB) is the leading cause of perinatal mortality and short- and long-term morbidity [1,2]. It is defined as birth that occurs before 37 weeks gestation due to spontaneous onset of labour or preterm premature rupture of membranes (pPROM) [3,4]. Several pathways and mechanisms linked with preterm birth have been proposed including, neuroendocrine, vascular, immune-inflammatory, and behavioural processes [5]. More specifically, several markers associated with uterine distension/contraction, decidual inflammation/infection and activation of hypothalamic–pituitary–adrenal axis had been studied in the past decades [5,6]. However, no single marker or combination of markers has been found to be accurate enough for predicting sPTB [7–10].

Preterm birth is a complex and multifactorial syndrome that possibly has a long pre-clinical phase, maternal and fetal interactions, genetic and environmental influences, and adaptive mechanisms [11,12]. These challenging aspects, and the presence of still unknown underlying mechanisms, are the main limitations for the identification of an accurate predictor for sPTB [13–15]. None of the predictors used in clinical practice, such as previous history of preterm birth, infection (vaginal and urinary contaminants), fibronectin and transvaginal ultrasonography cervical length demonstrated exceptional accuracy for predicting spontaneous preterm birth [7]. An exploration of innovative approaches is urgently required.

Metabolomics is the study of metabolites, through identification and quantification of low-weight molecular particles, i.e. tens to hundreds thousands of intermediate products and substrates of systems biology chemical reactions [16,17]. This novel approach has been applied for identifying biomarkers and underlying biochemical pathways associated with complex obstetrical syndromes as preeclampsia, fetal growth restriction, gestational

For peer review only - http://bmjopen.bmj.com/site/about/guidelines.xhtml

diabetes and preterm birth. In contrast to other "*Omics Sciences*" techniques, metabolomics is more closely associated with the phenotype of the disease and might thus identify a more robust and reliable set of predictors [18]. Importantly, implementing an adequate *Omics* experimental design is crucial for metabolomics studies.

Using different baseline population (asymptomatic vs symptomatic or low- vs high-risk women for developing sPTB), study designs (prospective cohorts, case-control or cross sectional studies), sources of samples (amniotic fluid, vaginal fluid, blood, urine, hair, etc) and the timing of sample collection each have significant effects on study findings and the consequent interpretation and contribution to the current gap of knowledge [16]. Therefore, we aim to conduct a systematic review of the use of metabolomics biomarkers for predicting spontaneous preterm birth in asymptomatic pregnant women. This protocol describes the methods that will be applied in our systematic review.

METHODS AND ANALYSIS

The current systematic review proposal will be conducted, written and published following the Preferred Reporting Items for Systematics Reviews and Meta-Analyses (PRISMA-P) recommendations [19]. Also, it is properly registered at PROSPERO platform – code CRD42018100172.

Review question

What is the performance of metabolomics for predicting spontaneous preterm birth in asymptomatic pregnant women?

Eligibility Criteria

Original cohort or case-control studies involving asymptomatic pregnant women at the moment of sample collection (exposure) and with samples analysed using metabolomics

For peer review only - http://bmjopen.bmj.com/site/about/guidelines.xhtml

techniques. Studies will be excluded if (1) they are cross-sectional studies, clinical trials, editorials, letter to editors, case reports, expert opinions, commentaries, or any type of review; (2) they describes only experimental studies with animals; or (3) they are duplicated data (e.g. data published in conferences proceedings and, then, published again in scientific journals). In this case, only the most complete publication will be considered, after comparing and confirming that the same technique and metabolites were explored. Studies published from 2008 to 2018 will be considered, and there will be no language restriction. Before submitting this systematic review for publication, we will rerun the search strategy to identify new studies that have been published after performing the first round of search.

Participants

The current review is interested in evaluating the performance of metabolomics biomarkers for spontaneous preterm birth in asymptomatic women, which may contribute to clinical practice, potentially providing information regarding onset of preterm labour. Nevertheless, we aim to identify studies addressing only early predictors collected from asymptomatic women (i.e. women who are in an early preclinical stage), which might contribute to a wider window of opportunity for interventions and also to develop a widely reproducible screening test. Asymptomatic pregnant women should not have regular uterine tightening/contractions or signs of rupture of membranes (i.e. watery discharge). In addition, the study should preferably have a standardized definition of spontaneous preterm birth, the outcome of interest.

Information Sources

The search will be held in the following databases: PubMed, EMBASE, ProQuest, Scopus, CINAHL, and Web of Science, BVS/BIREME, which includes the Latin American and Caribbean Health Sciences Literature (LILACS), Medline and the Scientific Electronic Library

For peer review only - http://bmjopen.bmj.com/site/about/guidelines.xhtml

Online (Scielo). In addition, secondary sources of original studies will be explored such as Google Scholar, hand-held searching of the reference list of eligible studies, conference proceedings, and contact with authors when necessary.

Search Strategy

The following terms will be used in our search strategy for the different scientific databases: (preterm birth, premature birth, premature infant, premature labor, extremely premature infant, premature obstetric labor, spontaneous preterm birth, extreme preterm birth, late preterm birth, moderate preterm birth, preterm premature rupture of membranes, preterm delivery, PROM, sPTB, preterm PROM, pPROM, p-PROM) AND (metabolomic*, metabonomic*, metabolit*, lipidomic*, H NMR, proton NMR, proton nuclear magnetic resonance, liquid chromatogra*, gas chromatogra*, UPLC, ultra-performance liquid chromatograph*, ultra performance liquid chromatograph*, HPLC, high performance liquid chrormatograph*, high-performance liquid chrormatograph*) AND (pregnan*, antenat*, ante nat*, prenat*, pre nat*). Respective adaptations in the syntax of search for each database will be applied accordingly. No filters - such as “research in animal’s models” and “reviews” - will be used in our search strategy, as it will be excluded according to eligibility criteria. The complete search strategy, including Boolean terms, is provided as Supplementary Material.

Data Management

We will export search results to a reference manager (Mendeley®). Then, the following information will be collected from each study using an appropriate form, which will be entered in an Excel® spreadsheet: author’s name, year of publication, country, study design, number of participants with and without spontaneous preterm birth, type of metabolomics analysis technique (liquid or gas chromatography, nuclear resonance), laboratory methods

For peer review only - http://bmjopen.bmj.com/site/about/guidelines.xhtml

for metabolites data acquiring (targeted or untargeted techniques, etc), subtype of preterm birth (spontaneous or pPROM), number of fetuses (singleton vs multiple), gestational age when samples were collected, source of samples (type/site of tissue), low or high-risk for preterm birth (authors criteria used to define the population will be collected) and method applied to estimate gestational age. If possible, additional variables related to spontaneous preterm birth categories (delivery before 28 weeks and before 34 weeks) will be recorded for secondary analyses. Finally, we will check the biochemical class of identified metabolites in Human Metabolome Database (HMDB, version 4.0) to explore and synthetize whether there are common biological pathways associated with spontaneous preterm birth [17].

Selection Process

Two independent reviewers (RTS and RBFG) will be responsible for screening and selecting studies initially according to title or abstract. Full text of non-excluded studies will be read to discriminate eligibility. A third reviewer (DFBL) will consider any disagreement; additional reviewers (RPJ, PNB and JGC) will be responsible for supervising all steps and approving data extraction.

Data Collection Process

We will extract search results to a reference manager where all studies will be stored. Then, included studies will be placed in a new folder. Finally, we will manually extract data of interest from these included studies to an Excel® file. Each reviewer will have their own reference manager account, file and folder and discrepant results will be discussed together with the third reviewer.

Outcomes and Prioritization

For peer review only - http://bmjopen.bmj.com/site/about/guidelines.xhtml

The primary outcome is spontaneous preterm birth, defined as any birth occurred before 37 weeks of gestation due to spontaneous onset of labor or preterm premature rupture of membranes. Secondary outcomes are:

1) Spontaneous preterm birth before 28 weeks;
2) Spontaneous preterm birth before 34 weeks;

The capacity to predict different degrees of sPTB (categories of gestational age) is important as the extreme (<28wks) and non-late preterm (<34wks) newborns have different adverse outcomes compared to non-extreme (≥28wks) or late (≥34 wks) preterm newborns.

Ideally, the method of gestational age estimation should be clearly reported. For instance, it can be reported as estimated by last menstrual period (LMP) and confirmed by an early ultrasound or only by an early ultrasound when LMP is unknown/uncertain.

Index test

Metabolomics techniques to predict spontaneous preterm birth is the diagnostic test of interest. Metabolomics is a technique to identify and quantify metabolites from biological samples using different type of platforms/equipment. The most common platforms include gas, liquid chromatography or ultra-performance liquid chromatography coupled to a mass spectrometer or a proton nuclear magnetic resonance [20]. If possible, the performance of each metabolomics techniques will be assessed through hierarchical summary receiver operator characteristic curve (HSROC) (meta-analysis).

Risk of Bias in individual Studies

We will apply the Quality Assessment of Diagnostic Accuracy Studies (QUADAS-2) tool [21] to assess the risk of bias and applicability of primary diagnostic accuracy studies. Each study will be classified as “low”, “high” or “unclear” regarding risk of bias for each of the four domains of QUADAS tool: Patient Selection, Index Test (metabolomics), Reference Standard

(occurrence of preterm birth), and Flow and Timing of participant's inclusion and follow-up. For example, studies will be labelled as "low" risk of bias for Reference Standard when definition of spontaneous preterm birth and gestational age estimation are clear; "high" risk of bias would be considered when the moment of sample collection is not well described.

Data Synthesis

We will report details of identification, screening, eligibility and included studies using a flow diagram, according to PRISMA recommendations [19]. Data from included studies will be synthetized into tables according to the variables of interest. If possible, we will present data meta-analysis according to study design, metabolomics technique and type of samples analysed. We intend to perform subgroup analysis according to:

- Different metabolomics methods applied: gas or liquid chromatography coupled with mass spectrometry or proton nuclear magnetic resonance;
- Singleton and multiple pregnancies;
- Low-risk and high-risk women for developing preterm birth;
- Subtype of preterm birth: Spontaneous preterm birth exclusively due to spontaneous onset of labour with intact membranes or sPTB due to premature rupture of membranes.

Potential anticipated limitations to this review

Firstly, although we have not considered any language restriction, we consider that there might be a limitation in studies published entirely in non-English language. However, in the last decade, more than 95% of scientific biomedical literature has been published in English [22], then we consider this a minor selection bias. Secondly, we intend to stratify the groups according to population risk. However, the characterization of low- or high-risk for spontaneous preterm birth is controversial and lacks standardization, which might limit data

For peer review only - http://bmjopen.bmj.com/site/about/guidelines.xhtml

comparison and subgroup analysis. Finally, categorization of sPTB into spontaneous onset of labour or pPROM is another topic of potential limitation - the recognition of the main initial mechanism for preterm delivery might not always be possible. Even when specified, it might provoke uncertainty and could limit further considerations regarding preterm phenotypes.

Patient and Public Involvement

Patients will not be directly involved in the study and no experience or direct impact from their perspective can be discussed.

ETHICS AND DISSEMINATION

This systematic review does not require ethical approval from the Research Council or Ethics board. We intend to disseminate our findings in scientific peer-reviewed journal, general free access website of Preterm Screening and Metabolomics in Brazil and Auckland (Preterm SAMBA) study, specialists' conferences, and to our funding agencies.

DISCUSSION

This systematic review will comprise current knowledge related with metabolomics in the context of preterm birth prediction. Metabolomics science, a resourceful innovative field that allows better understanding on pathophysiology of complex syndromes, may address the main compounds associated with the spontaneous preterm delivery and, therefore, motivate further researchers to validate early measurable predictors of preterm birth.

Metabolomics performance for predicting sPTB remains unclear and standardized and high-quality studies are needed to clarify the clinical application of metabolites for predicting sPTB. Nevertheless, metabolomics discovery studies commonly requires further validation studies; reproducible methodology is crucial. This systematic review protocol will collate the main potential early biomarkers, subgroup analysis and standardized definition for

For peer review only - http://bmjopen.bmj.com/site/about/guidelines.xhtml

spontaneous preterm birth to better understand metabolomics performance in predicting sPTB and also to show its heterogeneity in terms of methodology (samples used, metabolomics technique, definition of SPTB phenotype, etc). High performing predictors of preterm birth will help combat this leading cause of neonatal mortality and morbidity.

References

1 Lawn JE, Cousens S, Zupan J. 4 million neonatal deaths: when? Where? Why? *Lancet* 2005;**365**:891–900. doi:10.1016/S0140-6736(05)71048-5

2 Howson CP, Kinney M V, McDougall L, *et al.* Born Too Soon: Preterm birth matters. *Reprod Health* 2013;**10 Suppl 1**:S1. doi:10.1186/1742-4755-10-S1-S1

3 Blencowe H, Cousens S, Chou D, *et al.* Born Too Soon: The global epidemiology of 15 million preterm births. *Reprod Health* 2013;**10**:S2. doi:10.1186/1742-4755-10-S1-S2

4 Ananth C V, Vintzileos AM. Epidemiology of preterm birth and its clinical subtypes. *J Matern Fetal Neonatal Med* 2006;**19**:773–82. doi:10.1080/14767050600965882

5 Behrman R, ButlerAS, editors. *Institute of Medicine (IOM). Preterm Birth: Causes, Consequences, and Prevention.* Washington, D.C.: : National Academies Press 2007. doi:10.17226/11622

6 Manuck TA, Esplin MS, Biggio J, *et al.* The phenotype of spontaneous preterm birth: application of a clinical phenotyping tool. *Am J Obstet Gynecol* Published Online First: February 2015. doi:10.1016/j.ajog.2015.02.010

7 Honest H, Hyde CJ, Khan KS. Prediction of spontaneous preterm birth: no good test for predicting a spontaneous preterm birth. *Curr Opin Obstet Gynecol* 2012;**24**:422–33. doi:10.1097/GCO.0b013e328359823a

8 Conde-Agudelo A, Papageorghiou A, Kennedy S, *et al.* Novel biomarkers for the prediction of the spontaneous preterm birth phenotype: a systematic review and meta-analysis. *BJOG An Int J Obstet Gynaecol* 2011;**118**:1042–54. doi:10.1111/j.1471-0528.2011.02923.x

9 Hee L. Likelihood ratios for the prediction of preterm delivery with biomarkers. *Acta Obstet Gynecol Scand* 2011;**90**:1189–99. doi:10.1111/j.1600-0412.2011.01187.x

10 Goldenberg RL, Iams JD, Mercer BM, *et al.* The Preterm Prediction Study: toward a multiple-marker test for spontaneous preterm birth. *Am J Obstet Gynecol* 2001;**185**:643–51. doi:10.1067/mob.2001.116752

11 Di Renzo GC. The great obstetrical syndromes. *J Matern neonatal Med* 2009;**22**:633–

For peer review only

For peer review only - http://bmjopen.bmj.com/site/about/guidelines.xhtml

5. doi:10.1080/14767050902866804

12 Brosens I, Pijnenborg R, Vercruysse L, *et al.* The ‘Great Obstetrical Syndromes’ are associated with disorders of deep placentation. *Am J Obstet Gynecol* 2011;**204**:193–201. doi:10.1016/j.ajog.2010.08.009

13 Practice bulletin no. 130: prediction and prevention of preterm birth. *Obstet Gynecol* 2012;**120**:964–73. doi:10.1097/AOG.0b013e3182723b1b

14 Goldenberg RL, Culhane JF, Iams JD, *et al.* Epidemiology and causes of preterm birth. *Lancet* 2008;**371**:75–84. doi:10.1016/S0140-6736(08)60074-4

15 Honest H, Hyde CJ, Khan KS. Prediction of spontaneous preterm birth. *Curr Opin Obstet Gynecol* 2012;**24**:422–33. doi:10.1097/GCO.0b013e328359823a

16 Horgan RP, Clancy OH, Myers JE, *et al.* An overview of proteomic and metabolomic technologies and their application to pregnancy research. *BJOG* 2009;**116**:173–81. doi:10.1111/j.1471-0528.2008.01997.x

17 Wishart DS, Feunang YD, Marcu A, *et al.* HMDB 4.0: the human metabolome database for 2018. *Nucleic Acids Res* 2018;**46**:D608–17. doi:10.1093/nar/gkx1089

18 Dettmer K, Hammock BD. Metabolomics--a new exciting field within the omics sciences. *Environ Health Perspect* 2004;**112**:A396-7.

19 Shamseer L, Moher D, Clarke M, *et al.* Preferred reporting items for systematic review and meta-analysis protocols (PRISMA-P) 2015: elaboration and explanation. *BMJ* 2015;**350**:g7647. doi:10.1136/BMJ.G7647

20 Zhang A, Sun H, Wang P, *et al.* Modern analytical techniques in metabolomics analysis. *Analyst* 2012;**137**:293–300. doi:10.1039/c1an15605e

21 Whiting PF, Rutjes AWS, Westwood ME, *et al.* QUADAS-2: A Revised Tool for the Quality Assessment of Diagnostic Accuracy Studies. *Ann Intern Med* 2011;**155**:529. doi:10.7326/0003-4819-155-8-201110180-00009

22 Rosselli D. The language of biomedical sciences. *Lancet* 2016;**387**:1720–1. doi:10.1016/S0140-6736(16)30259-8

Author´s Contributions

RTS and RBFG will conduct the systematic review as independent first reviewers. JGC, RPJ and DFBL will decide about conflicting decisions regarding papers selections. PNB, RPJ and

For peer review only

For peer review only - http://bmjopen.bmj.com/site/about/guidelines.xhtml

JGC participated in the systematic review conception, methodology and framework, together will all the others co-authors.

Funding

This research was supported by Brazilian National Research Council (grant number 401636/2013-5) and Bill and Melinda Gates Foundation (grant number OPP1107597- Grand Challenges Brazil: Reducing the burden of preterm birth, FIOTEC number 05/2013), which provided funding to PRETERM-SAMBA project (www.medscinet.com/samba). RTS and DFL have been awarded PhD scholarships from the CAPES Foundation, an agency under the Ministry of Education of Brazil, process 88881.134095/2016-01 and 8881.134512/2016-01 respectively. The sponsors played no role on the study design or manuscript writing.

Competing interests

All authors are carrying original research about metabolomics and presenting conferences about this topic, including spontaneous preterm birth, preeclampsia, gestational diabetes mellitus and fetal growth restriction. Philip N Baker is principal investigator of Metabolomics Diagnostics Ltd, a company dedicated to develop innovative screening tests using metabolomics technology.

Acknowledgements

Ana Paula de Morais e Oliveira, librarian of University of Campinas – Unicamp, Brazil, for collaborating in developing search strategy and Rachel Hanisch for her suggestions to some sections of the paper.

Ethics approval and consent to participate

For peer review only

For peer review only - http://bmjopen.bmj.com/site/about/guidelines.xhtml

This systematic review does not require ethical approval from the Research Council or Ethics board.

For peer review only

For peer review only - http://bmjopen.bmj.com/site/about/guidelines.xhtml

Seacrh strategy: #1 AND #2 AND #3

1 (OR for each term)	preterm birth<br>premature birth<br>premature infant<br>premature labor<br>extremely premature infant<br>premature obstetric labor<br>spontaneous preterm birth<br>extreme preterm birth<br>late preterm birth<br>moderate preterm birth<br>preterm premature rupture of membranes<br>preterm delivery<br>PROM<br>sPTB<br>preterm PROM<br>pPROM<br>p-PROM
2 (OR for each term)	metabolomic*<br>metabonomic*<br>metabolit*<br>lipidomic*<br>H NMR<br>proton NMR<br>proton nuclear magnetic resonance<br>liquid chromatogra*<br>UPLC<br>ultra-performance liquid chromatograph*<br>ultra performance liquid chromatograph*<br>HPLC<br>high performance liquid chromatograph*<br>high-performance liquid chromatograph*
3 (OR for each term)	pregnan*<br>antenat*<br>ante nat*<br>prenat*<br>pre nat*

For peer review only

PRISMA-P 2015 Checklist

This checklist has been adapted for use with systematic review protocol submissions to BioMed Central journals from Table 3 in Moher D et al**:** Preferred reporting items for systematic review and meta-analysis protocols (PRISMA-P) 2015 statement. *Systematic Reviews* 2015 **4**:1

An Editorial from the Editors-in-Chief of *Systematic Reviews* details why this checklist was adapted - Moher D, Stewart L & Shekelle P**:** Implementing PRISMA-P: recommendations for prospective authors. *Systematic Reviews* 2016 **5**:15

Section/topic	#	Checklist item	Information reported: Yes	Information reported: No	Line number(s)
**ADMINISTRATIVE INFORMATION**					
**Title**					
Identification	1a	Identify the report as a protocol of a systematic review	x	☐	2
Update	1b	If the protocol is for an update of a previous systematic review, identify as such	☐	☐	n/a
**Registration**	2	If registered, provide the name of the registry (e.g., PROSPERO) and registration number in the Abstract	x	☐	48-49
**Authors**					
Contact	3a	Provide name, institutional affiliation, and e-mail address of all protocol authors; provide physical mailing address of corresponding author	x	☐	4-23
Contributions	3b	Describe contributions of protocol authors and identify the guarantor of the review	x	☐	283-287
**Amendments**	4	If the protocol represents an amendment of a previously completed or published protocol, identify as such and list changes; otherwise, state plan for documenting important protocol amendments	☐	☐	n/a
**Support**					
Sources	5a	Indicate sources of financial or other support for the review	x	☐	275-282
Sponsor	5b	Provide name for the review funder and/or sponsor	x	☐	275-282
Role of sponsor/funder	5c	Describe roles of funder(s), sponsor(s), and/or institution(s), if any, in developing the protocol	x	☐	282
**INTRODUCTION**					
**Rationale**	6	Describe the rationale for the review in the context of what is already known	x	☐	51-87
**Objectives**	7	Provide an explicit statement of the question(s) the review will address with reference to	x	☐	94-95

For peer review only - http://bmjopen.bmj.com/site/about/guidelines.xhtml

2

Section/topic	#	Checklist item	Information reported Yes	Information reported No	Line number(s)
		participants, interventions, comparators, and outcomes (PICO)			
**METHODS**					
**Eligibility criteria**	8	Specify the study characteristics (e.g., PICO, study design, setting, time frame) and report characteristics (e.g., years considered, language, publication status) to be used as criteria for eligibility for the review	x	☐	96-107
**Information sources**	9	Describe all intended information sources (e.g., electronic databases, contact with study authors, trial registers, or other grey literature sources) with planned dates of coverage	x	☐	119-125
**Search strategy**	10	Present draft of search strategy to be used for at least one electronic database, including planned limits, such that it could be repeated	x	☐	127-136
STUDY RECORDS					
Data management	11a	Describe the mechanism(s) that will be used to manage records and data throughout the review	x	☐	142-155
Selection process	11b	State the process that will be used for selecting studies (e.g., two independent reviewers) through each phase of the review (i.e., screening, eligibility, and inclusion in meta-analysis)	x	☐	157-161
Data collection process	11c	Describe planned method of extracting data from reports (e.g., piloting forms, done independently, in duplicate), any processes for obtaining and confirming data from investigators	x	☐	163-167
**Data items**	12	List and define all variables for which data will be sought (e.g., PICO items, funding sources), any pre-planned data assumptions and simplifications	x	☐	142-155
**Outcomes and prioritization**	13	List and define all outcomes for which data will be sought, including prioritization of main and additional outcomes, with rationale	x	☐	169-179
**Risk of bias in individual studies**	14	Describe anticipated methods for assessing risk of bias of individual studies, including whether this will be done at the outcome or study level, or both; state how this information will be used in data synthesis	x	☐	189-196
DATA					
**Synthesis**	15a	Describe criteria under which study data will be quantitatively synthesized	x	☐	198-209
	15b	If data are appropriate for quantitative synthesis, describe planned summary measures, methods of handling data, and methods of combining data from studies, including any planned exploration of consistency (e.g., I^2, Kendall's tau)	x	☐	185-187
	15c	Describe any proposed additional analyses (e.g., sensitivity or subgroup analyses, meta-regression)	x	☐	185-187

For peer review only

Section/topic	#	Checklist item	Information reported		Line number(s)
			Yes	No	
	15d	If quantitative synthesis is not appropriate, describe the type of summary planned	x	☐	198-202
**Meta-bias(es)**	16	Specify any planned assessment of meta-bias(es) (e.g., publication bias across studies, selective reporting within studies)	☐	☐	n/a
**Confidence in cumulative evidence**	17	Describe how the strength of the body of evidence will be assessed (e.g., GRADE)	☐	☐	n/a

For peer review only

BMJ Open

The use of metabolomics for predicting spontaneous preterm birth in asymptomatic pregnant women: protocol for a systematic review and meta-analysis

Journal:	*BMJ Open*
Manuscript ID	bmjopen-2018-026033.R1
Article Type:	Protocol
Date Submitted by the Author:	16-Nov-2018
Complete List of Authors:	Souza, Renato; Universidade Estadual de Campinas, Obstetrics and Gynecology<br>Galvão, Rafael; Universidade Estadual de Campinas, Obstetrics and Gynecology<br>Leite, Debora; Campinas' State University, Department of Tocogynecology; Universidade Federal de Pernambuco,<br>Passini Jr, Renato; Universidade Estadual de Campinas Faculdade de Ciencias Medicas<br>Baker, Philip ; University of Leicester, College of Medicine<br>Cecatti, Jose; University of Campinas, Obstetrics and Gynecology
**Primary Subject Heading**:	Obstetrics and gynaecology
Secondary Subject Heading:	Diagnostics
Keywords:	preterm birth, metabolomics, biomarkers, prediction

SCHOLARONE™
Manuscripts

The use of metabolomics for predicting spontaneous preterm birth in asymptomatic pregnant women: protocol for a systematic review and meta-analysis

Renato T. Souza [1] (renatotsouzasp@gmail.com)

Rafael Bessa Freitas Galvão [1] (rafaelbfg@gmail.com)

Débora Farias Batista Leite [1,2] (deborafariasleite@gmail.com)

Renato Passini Jr [1] (passini@caism.unicamp.br)

Philip N. Baker [3] (philip.baker@leicester.ac.uk)

José Guilherme Cecatti [1] (cecatti@unicamp.br)

[1] Department of Obstetrics and Gynaecology, University of Campinas, Campinas, São Paulo, Brazil.

[2] Clinics Hospital of Federal University of Pernambuco, Recife, Pernambuco, Brazil.

[3] College of Life Sciences, University of Leicester, England, United Kingdom.

This systematic review protocol is registered at PROSPERO platform (CRD42018100172). Available from: http://www.crd.york.ac.uk/PROSPERO/display_record.php?ID=CRD42018100172.

Corresponding author:

José Guilherme Cecatti

101 Alexander Fleming, Cidade Universitária, campinas, São Paulo, Brazil.

ZIPCODE 13083-881

cecatti@unicamp.br

Word count: 2,233.

For peer review only

ABSTRACT

Introduction: Preterm birth (PTB) is the leading cause of neonatal mortality and short- and long-term morbidity. The aetiology and pathophysiology of spontaneous PTB are still unclear, which makes the identification of reliable and accurate predictor markers more difficult, particularly for unscreened or asymptomatic women. Metabolomics biomarkers have been demonstrated to be potentially accurate biomarkers for many disorders with complex mechanisms such as PTB. Therefore, we aim to perform a systematic review of metabolomics markers associated with spontaneous PTB. Our research question is "What is the performance of metabolomics for predicting spontaneous preterm birth in asymptomatic pregnant women?"

Methods and analysis: We will focus on studies assessing metabolomics techniques for predicting spontaneous preterm birth in asymptomatic pregnant women. We will conduct a comprehensive systematic review of the literature from the last 10 years. Only observational cohort and case-control studies will be included. Our search strategy will be carried out by two independent reviewers, who will scan title and abstract before carrying out a full review of the article. The scientific databases to be explored include PubMed, MedLine, ScieLo, EMBASE, LILACS, Web of Science, Scopus, and others.

Ethics and dissemination: This systematic review protocol does not require ethical approval. We intend to disseminate our findings in scientific peer-reviewed journal, the Preterm SAMBA study open access website, specialists' conferences, and to our funding agencies.

Registration details: This protocol is registered in PROSPERO platform (code CRD42018100172).

Keywords: preterm birth, spontaneous preterm birth, metabolomics, biomarkers, prediction.

For peer review only - http://bmjopen.bmj.com/site/about/guidelines.xhtml

Strengths and limitations of this study

- This systematic review protocol takes into account some important aspects regarding conducting a systematic review about spontaneous preterm birth and metabolomics such as the criteria used for defining spontaneous preterm birth, different population risk stratification, method used to estimate gestational age, and metabolomics techniques details.
- Two independent reviewers are responsible for searching and selecting studies, as also extracting data, and a third reviewer will resolve any disagreement.
- If possible, proper statistical methods will be applied to investigate metabolomics accuracy in predicting spontaneous preterm birth.
- Possible limitations to this review include the different criteria applied for defining spontaneous preterm birth, and the diverse population risk stratification.

For peer review only

For peer review only - http://bmjopen.bmj.com/site/about/guidelines.xhtml

INTRODUCTION

Spontaneous preterm birth (sPTB) is the leading cause of perinatal mortality and short- and long-term morbidity [1,2]. It is defined as birth that occurs before 37 weeks gestation due to spontaneous onset of labour or preterm premature rupture of membranes (pPROM) [3,4]. Several pathways and mechanisms linked with preterm birth have been proposed including, neuroendocrine, vascular, immune-inflammatory, and behavioural processes [5]. More specifically, several markers associated with uterine distension/contraction, decidual inflammation/infection and activation of hypothalamic–pituitary–adrenal axis had been studied in the past decades [5,6]. However, no single marker or combination of markers has been found to be accurate enough for predicting sPTB [7–10]. History of previous preterm birth, cervical length at second trimester and cervico-vaginal fetal fibronectin are the most promising clinical tests for predicting spontaneous preterm, but they seem not to be clinically useful for asymptomatic women. Sensitivity of short cervical length (<25mm) and high cervico-vaginal fFN (>50ng/ml) are around 33-36% and 46%, respectively [11–13].

Preterm birth is a complex and multifactorial syndrome that possibly has a long pre-clinical phase, maternal and fetal interactions, genetic and environmental influences, and adaptive mechanisms [14,15]. These challenging aspects, and the presence of still unknown underlying mechanisms, are the main limitations for the identification of an accurate predictor for sPTB [16–18]. None of the predictors used in clinical practice, such as previous history of preterm birth, infection (vaginal and urinary contaminants), fibronectin and transvaginal ultrasonography cervical length demonstrated exceptional accuracy for predicting spontaneous preterm birth [7]. An exploration of innovative approaches is urgently required.

For peer review only

Metabolomics is the study of metabolites, through identification and quantification of low-weight molecular particles, i.e. tens to hundreds thousands of intermediate products and substrates of systems biology chemical reactions [19,20]. This novel approach has been applied for identifying biomarkers and underlying biochemical pathways associated with complex obstetrical syndromes as preeclampsia, fetal growth restriction, gestational diabetes and preterm birth. In contrast to other "*Omics Sciences*" techniques, metabolomics is more closely associated with the phenotype of the disease and might thus identify a more robust and reliable set of predictors [21]. Importantly, implementing an adequate *Omics* experimental design is crucial for metabolomics studies. Using different baseline population (asymptomatic vs symptomatic or low- vs high-risk women for developing sPTB), study designs (prospective cohorts, case-control or cross sectional studies), sources of samples (amniotic fluid, vaginal fluid, blood, urine, hair, etc) and the timing of sample collection each have significant effects on study findings and the consequent interpretation and contribution to the current gap of knowledge [19].

Different reviews collating scientific knowledge regarding preterm birth biomarkers/predictors has been conducted. Different methodology approaches has been applied so far, including narrative, systematic and umbrella reviews, a more comprehensive review that includes not only original studies but also other reviews [7,19,22,23]. At the best of our knowledge, there is no systematic review on metabolomics markers. Therefore, we aim to conduct a systematic review of original studies investigating the use of metabolomics biomarkers for predicting spontaneous preterm birth in asymptomatic pregnant women. This protocol describes the methods that will be applied in our systematic review.

METHODS AND ANALYSIS

For peer review only

For peer review only - http://bmjopen.bmj.com/site/about/guidelines.xhtml

The current systematic review proposal will be conducted, written and published following the Preferred Reporting Items for Systematics Reviews and Meta-Analyses (PRISMA-P) recommendations [24]. Also, it is properly registered at PROSPERO platform – code CRD42018100172.

Review question

What is the performance of metabolomics for predicting spontaneous preterm birth in asymptomatic pregnant women?

Eligibility Criteria

Original cohort or case-control studies involving asymptomatic pregnant women at the moment of sample collection (exposure) and with samples analysed using metabolomics techniques. Studies will be excluded if (1) they are cross-sectional studies, clinical trials, editorials, letter to editors, case reports, expert opinions, commentaries, or any type of review; (2) they describes only experimental studies with animals; or (3) they are duplicated data (e.g. data published in conferences proceedings and, then, published again in scientific journals). In this case, only the most complete publication will be considered, after comparing and confirming that the same technique and metabolites were explored. Studies published from 2008 to 2018 will be considered, and there will be no language restriction. Before submitting this systematic review for publication, we will rerun the search strategy to identify new studies that have been published after performing the first round of search.

Participants

The current review is interested in evaluating the performance of metabolomics biomarkers for spontaneous preterm birth in asymptomatic pregnant women, which may contribute to clinical practice, potentially providing information regarding onset of preterm labour. Nevertheless, we aim to identify studies addressing only early predictors collected from

For peer review only - http://bmjopen.bmj.com/site/about/guidelines.xhtml

women who are in an early preclinical stage, which might contribute to a wider window of opportunity for interventions and also to develop a widely reproducible screening test. Asymptomatic pregnant women should not have regular uterine tightening/contractions or signs of rupture of membranes (i.e. watery discharge). In addition, the study should preferably have a standardized definition of spontaneous preterm birth, the outcome of interest.

Information Sources

The search will be held in the following databases: PubMed, EMBASE, Scopus, CINAHL, and Web of Science, BVS/BIREME, which includes the Latin American and Caribbean Health Sciences Literature (LILACS), Medline and the Scientific Electronic Library Online (Scielo). In addition, secondary sources of original studies will be explored such as Google Scholar, hand-held searching of the reference list of eligible studies, conference proceedings, and contact with authors when necessary.

Search Strategy

The following terms will be used in our search strategy for the different scientific databases: (preterm birth, premature birth, premature infant, premature labor, extremely premature infant, premature obstetric labor, spontaneous preterm birth, extreme preterm birth, late preterm birth, moderate preterm birth, preterm premature rupture of membranes, preterm delivery, PROM, sPTB, preterm PROM, pPROM, p-PROM) AND (metabolomic*, metabonomic*, metabolit*, lipidomic*, H NMR, proton NMR, proton nuclear magnetic resonance, liquid chromatogra*, gas chromatogra*, UPLC, ultra-performance liquid chromatograph*, ultra-performance liquid chromatograph*, HPLC, high performance liquid chrormatograph*, high-performance liquid chrormatograph*) AND (pregnan*, antenat*, ante nat*, prenat*, pre nat*) (Supplementary Material). Respective adaptations in the

For peer review only - http://bmjopen.bmj.com/site/about/guidelines.xhtml

syntax of search for each database will be applied accordingly. No filters - such as “research in animal’s models” and “reviews” - will be used in our search strategy, as it will be excluded according to eligibility criteria. The complete search strategy, including Boolean terms, is provided as Supplementary Material.

Data Management

We will export search results to a reference manager (Mendeley®). Then, the following information will be collected from each study using an appropriate form, which will be entered in an Excel® spreadsheet: author’s name, year of publication, country, study design, number of participants with and without spontaneous preterm birth, type of metabolomics analysis technique (liquid or gas chromatography, nuclear resonance), laboratory methods for metabolites data acquiring (targeted or untargeted techniques, etc), subtype of preterm birth (spontaneous preterm labour or pPROM), number of fetuses (singleton vs multiple), gestational age when samples were collected, source of samples (type/site of tissue), low or high-risk for preterm birth (authors criteria used to define the population will be collected) and method applied to estimate gestational age. If possible, additional variables related to spontaneous preterm birth categories (delivery before 28 weeks and before 34 weeks) will be recorded for secondary analyses. Original authors will be contacted to clarify data, when needed. Finally, we will check the biochemical class of identified metabolites in Human Metabolome Database (HMDB, version 4.0) to explore and synthetize whether there are common biological pathways associated with spontaneous preterm birth [20].

Selection Process

Two independent reviewers (RTS and RBFG) will be responsible for screening and selecting studies initially according to title or abstract. Both researchers will read the full text of non-excluded studies to discriminate eligibility. A third reviewer (DFBL) will consider any

For peer review only - http://bmjopen.bmj.com/site/about/guidelines.xhtml

disagreement; additional reviewers (RPJ, PNB and JGC) will be responsible for supervising all steps and approving data extraction.

Data Collection Process

We will extract search results to a reference manager where all studies will be stored. Then, included studies will be placed in a new folder. Finally, we will manually extract data of interest from these included studies to an Excel® file. Each reviewer will have their own reference manager account, file and folder and discrepant results will be discussed together with the third reviewer.

Outcomes and Prioritization

The primary outcome is spontaneous preterm birth, defined as any birth occurred before 37 weeks of gestation due to spontaneous onset of labor or preterm premature rupture of membranes. Secondary outcomes are:

1. Spontaneous preterm birth before 28 weeks;
2. Spontaneous preterm birth before 32 weeks;
3. Spontaneous preterm birth before 34 weeks;

The capacity to predict different degrees of sPTB (categories of gestational age) is important as the extreme (<28wks), moderate (<32 weeks) and non-late preterm (<34wks) newborns have different adverse outcomes compared to non-extreme (≥28wks); non-moderate (≥32wks) or late (≥34 wks) preterm newborns.

Ideally, the method of gestational age estimation should be clearly reported. For instance, it can be reported as estimated by last menstrual period (LMP) and confirmed by an early ultrasound or only by an early ultrasound when LMP is unknown/uncertain.

Index test

For peer review only - http://bmjopen.bmj.com/site/about/guidelines.xhtml

Metabolomics techniques to predict spontaneous preterm birth is the diagnostic test of interest. Metabolomics is a technique to identify and quantify metabolites from biological samples using different type of platforms/equipment. The most common platforms include gas, liquid chromatography or ultra-performance liquid chromatography coupled to a mass spectrometer or a proton nuclear magnetic resonance [25]. If possible, the performance of each metabolomics techniques will be assessed through hierarchical summary receiver operator characteristic curve (HSROC) (meta-analysis).

Risk of Bias in individual Studies

We will apply the Quality Assessment of Diagnostic Accuracy Studies (QUADAS-2) tool [26] to assess the risk of bias and applicability of primary diagnostic accuracy studies. Each study will be classified as “low”, “high” or “unclear” regarding risk of bias for each of the four domains of QUADAS tool: Patient Selection, Index Test (metabolomics), Reference Standard (occurrence of preterm birth), and Flow and Timing of participant’s inclusion and follow-up. For example, studies will be labelled as “low” risk of bias for Reference Standard when definition of spontaneous preterm birth and gestational age estimation are clear; “high” risk of bias would be considered when the moment of sample collection is not well described.

Data Synthesis

We will report details of identification, screening, eligibility and included studies using a flow diagram, according to PRISMA recommendations [24]. Data from included studies will be synthetized into tables according to the variables of interest. If possible, we will present data meta-analysis according to study design, metabolomics technique and type of samples analysed. We intend to perform subgroup analysis according to:

- Different metabolomics methods applied: gas or liquid chromatography coupled with mass spectrometry or proton nuclear magnetic resonance;

For peer review only - http://bmjopen.bmj.com/site/about/guidelines.xhtml

- Singleton and multiple pregnancies;
- Low-risk and high-risk women for developing preterm birth;
- Subtype of preterm birth: Spontaneous preterm birth exclusively due to spontaneous onset of labour with intact membranes or sPTB due to premature rupture of membranes.

Heterogeneity will be assessed by Cochran's Q, Hotelling's T-squared (τ^2) and I^2 tests. Funnel plots and sensitivity and cumulative analyses will be applied for detection of temporal trends and publication bias.

Potential anticipated limitations to this review

Firstly, although we have not considered any language restriction, we consider that there might be a limitation in studies published entirely in non-English language. However, in the last decade, more than 95% of scientific biomedical literature has been published in English [27], then we consider this a minor selection bias. Secondly, we intend to stratify the groups according to population risk. However, the characterization of low- or high-risk for spontaneous preterm birth is controversial and lacks standardization, which might limit data comparison and subgroup analysis. Finally, categorization of sPTB into spontaneous onset of labour or pPROM is another topic of potential limitation - the recognition of the main initial mechanism for preterm delivery might not always be possible. Even when specified, it might provoke uncertainty and could limit further considerations regarding preterm phenotypes. In addition, another limitation is that individual patient data will not be collected.

Patient and Public Involvement

Patients will not be directly involved in the study and no experience or direct impact from their perspective can be discussed.

For peer review only - http://bmjopen.bmj.com/site/about/guidelines.xhtml

ETHICS AND DISSEMINATION

This systematic review does not require ethical approval from the Research Council or Ethics board. We intend to disseminate our findings in scientific peer-reviewed journal, general free access website of Preterm Screening and Metabolomics in Brazil and Auckland (Preterm SAMBA) study, specialists' conferences, and to our funding agencies.

DISCUSSION

This systematic review will comprise current knowledge related with metabolomics in the context of preterm birth prediction. Metabolomics science, a resourceful innovative field that allows better understanding on pathophysiology of complex syndromes, may address the main compounds associated with the spontaneous preterm delivery and, therefore, motivate further researchers to validate early measurable predictors of preterm birth.

Metabolomics performance for predicting sPTB remains unclear and standardized and high-quality studies are needed to clarify the clinical application of metabolites for predicting sPTB. Nevertheless, metabolomics discovery studies commonly requires further validation studies; reproducible methodology is crucial. This systematic review protocol will collate the main potential early biomarkers, subgroup analysis and standardized definition for spontaneous preterm birth to better understand metabolomics performance in predicting sPTB and also to show its heterogeneity in terms of methodology (samples used, metabolomics technique, definition of SPTB phenotype, etc). High performing predictors of preterm birth will help combat this leading cause of neonatal mortality and morbidity.

For peer review only - http://bmjopen.bmj.com/site/about/guidelines.xhtml

References

1. Lawn JE, Cousens S, Zupan J. 4 million neonatal deaths: when? Where? Why? *Lancet* 2005;**365**:891–900. doi:10.1016/S0140-6736(05)71048-5

2. Howson CP, Kinney M V, McDougall L, *et al.* Born Too Soon: Preterm birth matters. *Reprod Health* 2013;**10 Suppl 1**:S1. doi:10.1186/1742-4755-10-S1-S1

3. Blencowe H, Cousens S, Chou D, *et al.* Born Too Soon: The global epidemiology of 15 million preterm births. *Reprod Health* 2013;**10**:S2. doi:10.1186/1742-4755-10-S1-S2

4. Ananth C V, Vintzileos AM. Epidemiology of preterm birth and its clinical subtypes. *J Matern Fetal Neonatal Med* 2006;**19**:773–82. doi:10.1080/14767050600965882

5. Behrman R, ButlerAS, editors. *Institute of Medicine (IOM). Preterm Birth: Causes, Consequences, and Prevention.* Washington, D.C.: National Academies Press 2007. doi:10.17226/11622

6. Manuck TA, Esplin MS, Biggio J, *et al.* The phenotype of spontaneous preterm birth: application of a clinical phenotyping tool. *Am J Obstet Gynecol* 2015;**212**(4):487.e1-487.e11. doi:10.1016/j.ajog.2015.02.010

7. Honest H, Hyde CJ, Khan KS. Prediction of spontaneous preterm birth: no good test for predicting a spontaneous preterm birth. *Curr Opin Obstet Gynecol* 2012;**24**:422–33. doi:10.1097/GCO.0b013e328359823a

8. Conde-Agudelo A, Papageorghiou A, Kennedy S, *et al.* Novel biomarkers for the prediction of the spontaneous preterm birth phenotype: a systematic review and meta-analysis. *BJOG* 2011;**118**:1042–54. doi:10.1111/j.1471-0528.2011.02923.x

9. Hee L. Likelihood ratios for the prediction of preterm delivery with biomarkers. *Acta Obstet Gynecol Scand* 2011;**90**:1189–99. doi:10.1111/j.1600-0412.2011.01187.x

10. Goldenberg RL, Iams JD, Mercer BM, *et al.* The Preterm Prediction Study: toward a multiple-marker test for spontaneous preterm birth. *Am J Obstet Gynecol* 2001;**185**:643–51. doi:10.1067/mob.2001.116752

11. Iams JD, Goldenberg RL, Meis PJ, *et al.* The length of the cervix and the risk of spontaneous premature delivery. National Institute of Child Health and Human Development Maternal Fetal Medicine Unit Network. *N Engl J Med* 1996;**334**:567–72. doi:10.1056/NEJM199602293340904

12. Smith V, Devane D, Begley CM, *et al.* A systematic review and quality assessment of systematic reviews of fetal fibronectin and transvaginal length for predicting preterm birth. *Eur J Obstet Gynecol Reprod Biol* 2007;**133**:134–42. doi:10.1016/j.ejogrb.2007.03.005

13. Abbott DS, Hezelgrave NL, Seed PT, *et al.* Quantitative fetal fibronectin to predict preterm birth in asymptomatic women at high risk. *Obstet Gynecol* 2015;**125**:1168–

For peer review only

For peer review only - http://bmjopen.bmj.com/site/about/guidelines.xhtml

76. doi:10.1097/AOG.0000000000000754

14 Di Renzo GC. The great obstetrical syndromes. *J Matern neonatal Med* 2009;**22**:633–5. doi:10.1080/14767050902866804

15 Brosens I, Pijnenborg R, Vercruysse L, *et al.* The 'Great Obstetrical Syndromes' are associated with disorders of deep placentation. *Am J Obstet Gynecol* 2011;**204**:193–201. doi:10.1016/j.ajog.2010.08.009

16 Practice bulletin no. 130: prediction and prevention of preterm birth. *Obstet Gynecol* 2012;**120**:964–73. doi:10.1097/AOG.0b013e3182723b1b

17 Goldenberg RL, Culhane JF, Iams JD, *et al.* Epidemiology and causes of preterm birth. *Lancet* 2008;**371**:75–84. doi:10.1016/S0140-6736(08)60074-4

18 Honest H, Hyde CJ, Khan KS. Prediction of spontaneous preterm birth. *Curr Opin Obstet Gynecol* 2012;**24**:422–33. doi:10.1097/GCO.0b013e328359823a

19 Horgan RP, Clancy OH, Myers JE, *et al.* An overview of proteomic and metabolomic technologies and their application to pregnancy research. *BJOG* 2009;**116**:173–81. doi:10.1111/j.1471-0528.2008.01997.x

20 Wishart DS, Feunang YD, Marcu A, *et al.* HMDB 4.0: the human metabolome database for 2018. *Nucleic Acids Res* 2018;**46**:D608–17. doi:10.1093/nar/gkx1089

21 Dettmer K, Hammock BD. Metabolomics--a new exciting field within the omics sciences. *Environ Health Perspect* 2004;**112**:A396-7.

22 Lucaroni F, Morciano L, Rizzo G, *et al.* Biomarkers for predicting spontaneous preterm birth: an umbrella systematic review. *J Matern Neonatal Med* 2018;**31**:726–34. doi:10.1080/14767058.2017.1297404

23 Romero R, Espinoza J, Gotsch F, *et al.* The use of high-dimensional biology (genomics, transcriptomics, proteomics, and metabolomics) to understand the preterm parturition syndrome. *BJOG* 2006;**113**:118–35. doi:10.1111/j.1471-0528.2006.01150.x

24 Shamseer L, Moher D, Clarke M, *et al.* Preferred reporting items for systematic review and meta-analysis protocols (PRISMA-P) 2015: elaboration and explanation. *BMJ* 2015;**350**:g7647. doi:10.1136/BMJ.G7647

25 Zhang A, Sun H, Wang P, *et al.* Modern analytical techniques in metabolomics analysis. *Analyst* 2012;**137**:293–300. doi:10.1039/c1an15605e

26 Whiting PF, Rutjes AWS, Westwood ME, *et al.* QUADAS-2: A Revised Tool for the Quality Assessment of Diagnostic Accuracy Studies. *Ann Intern Med* 2011;**155**:529. doi:10.7326/0003-4819-155-8-201110180-00009

27 Rosselli D. The language of biomedical sciences. *Lancet* 2016;**387**:1720–1. doi:10.1016/S0140-6736(16)30259-8

For peer review only - http://bmjopen.bmj.com/site/about/guidelines.xhtml

Author´s Contributions

RTS and RBFG will conduct the systematic review as independent first reviewers. JGC, RPJ and DFBL will decide about conflicting decisions regarding papers selections. PNB, RPJ and JGC participated in the systematic review conception, methodology and framework, together will all the others co-authors.

Funding

This research was supported by Brazilian National Research Council (grant number 401636/2013-5) and Bill and Melinda Gates Foundation (grant number OPP1107597- Grand Challenges Brazil: Reducing the burden of preterm birth, FIOTEC number 05/2013), which provided funding to PRETERM-SAMBA project (www.medscinet.com/samba). RTS and DFL have been awarded PhD scholarships from the CAPES Foundation, an agency under the Ministry of Education of Brazil, process 88881.134095/2016-01 and 8881.134512/2016-01 respectively. The sponsors played no role on the study design or manuscript writing.

Competing interests

All authors are carrying original research about metabolomics and presenting conferences about this topic, including spontaneous preterm birth, preeclampsia, gestational diabetes mellitus and fetal growth restriction. Philip N Baker is principal investigator of Metabolomics Diagnostics Ltd, a company dedicated to develop innovative screening tests using metabolomics technology.

Acknowledgements

For peer review only

For peer review only - http://bmjopen.bmj.com/site/about/guidelines.xhtml

366 Ana Paula de Morais e Oliveira, librarian of University of Campinas – Unicamp, Brazil, for collaborating in developing search strategy and Rachel Hanisch for her suggestions to some sections of the paper.

Ethics approval and consent to participate

This systematic review does not require ethical approval from the Research Council or Ethics board.

For peer review only

For peer review only - http://bmjopen.bmj.com/site/about/guidelines.xhtml

Seacrh strategy: #1 AND #2 AND #3

1 (OR for each term)	preterm birth<br>premature birth<br>premature infant<br>premature labor<br>extremely premature infant<br>premature obstetric labor<br>spontaneous preterm birth<br>extreme preterm birth<br>late preterm birth<br>moderate preterm birth<br>preterm premature rupture of membranes<br>preterm delivery<br>PROM<br>sPTB<br>preterm PROM<br>pPROM<br>p-PROM
2 (OR for each term)	metabolomic*<br>metabonomic*<br>metabolit*<br>lipidomic*<br>H NMR<br>proton NMR<br>proton nuclear magnetic resonance<br>liquid chromatogra*<br>UPLC<br>ultra-performance liquid chromatograph*<br>ultra performance liquid chromatograph*<br>HPLC<br>high performance liquid chromatograph*<br>high-performance liquid chromatograph*
3 (OR for each term)	pregnan*<br>antenat*<br>ante nat*<br>prenat*<br>pre nat*

For peer review only

PRISMA-P 2015 Checklist

This checklist has been adapted for use with systematic review protocol submissions to BioMed Central journals from Table 3 in Moher D et al**:** Preferred reporting items for systematic review and meta-analysis protocols (PRISMA-P) 2015 statement. *Systematic Reviews* 2015 **4**:1

An Editorial from the Editors-in-Chief of *Systematic Reviews* details why this checklist was adapted - Moher D, Stewart L & Shekelle P**:** Implementing PRISMA-P: recommendations for prospective authors. *Systematic Reviews* 2016 **5**:15

Section/topic	#	Checklist item	Information reported		Line number(s)
			Yes	No	
**ADMINISTRATIVE INFORMATION**					
**Title**					
Identification	1a	Identify the report as a protocol of a systematic review	x		2
Update	1b	If the protocol is for an update of a previous systematic review, identify as such			n/a
**Registration**	2	If registered, provide the name of the registry (e.g., PROSPERO) and registration number in the Abstract	x		48-49
**Authors**					
Contact	3a	Provide name, institutional affiliation, and e-mail address of all protocol authors; provide physical mailing address of corresponding author	x		4-23
Contributions	3b	Describe contributions of protocol authors and identify the guarantor of the review	x		283-287
**Amendments**	4	If the protocol represents an amendment of a previously completed or published protocol, identify as such and list changes; otherwise, state plan for documenting important protocol amendments			n/a
**Support**					
Sources	5a	Indicate sources of financial or other support for the review	x		275-282
Sponsor	5b	Provide name for the review funder and/or sponsor	x		275-282
Role of sponsor/funder	5c	Describe roles of funder(s), sponsor(s), and/or institution(s), if any, in developing the protocol	x		282
**INTRODUCTION**					
**Rationale**	6	Describe the rationale for the review in the context of what is already known	x		51-87
**Objectives**	7	Provide an explicit statement of the question(s) the review will address with reference to	x		94-95

For peer review only

Section/topic	#	Checklist item	Information reported Yes	Information reported No	Line number(s)
		participants, interventions, comparators, and outcomes (PICO)			
**METHODS**					
**Eligibility criteria**	8	Specify the study characteristics (e.g., PICO, study design, setting, time frame) and report characteristics (e.g., years considered, language, publication status) to be used as criteria for eligibility for the review	x	☐	96-107
**Information sources**	9	Describe all intended information sources (e.g., electronic databases, contact with study authors, trial registers, or other grey literature sources) with planned dates of coverage	x	☐	119-125
**Search strategy**	10	Present draft of search strategy to be used for at least one electronic database, including planned limits, such that it could be repeated	x	☐	127-136
STUDY RECORDS					
Data management	11a	Describe the mechanism(s) that will be used to manage records and data throughout the review	x	☐	142-155
Selection process	11b	State the process that will be used for selecting studies (e.g., two independent reviewers) through each phase of the review (i.e., screening, eligibility, and inclusion in meta-analysis)	x	☐	157-161
Data collection process	11c	Describe planned method of extracting data from reports (e.g., piloting forms, done independently, in duplicate), any processes for obtaining and confirming data from investigators	x	☐	163-167
**Data items**	12	List and define all variables for which data will be sought (e.g., PICO items, funding sources), any pre-planned data assumptions and simplifications	x	☐	142-155
**Outcomes and prioritization**	13	List and define all outcomes for which data will be sought, including prioritization of main and additional outcomes, with rationale	x	☐	169-179
**Risk of bias in individual studies**	14	Describe anticipated methods for assessing risk of bias of individual studies, including whether this will be done at the outcome or study level, or both; state how this information will be used in data synthesis	x	☐	189-196
DATA					
**Synthesis**	15a	Describe criteria under which study data will be quantitatively synthesized	x	☐	198-209
	15b	If data are appropriate for quantitative synthesis, describe planned summary measures, methods of handling data, and methods of combining data from studies, including any planned exploration of consistency (e.g., I^2, Kendall's tau)	x	☐	185-187
	15c	Describe any proposed additional analyses (e.g., sensitivity or subgroup analyses, meta-regression)	x	☐	185-187

For peer review only

Section/topic	#	Checklist item	Information reported Yes	No	Line number(s)
	15d	If quantitative synthesis is not appropriate, describe the type of summary planned	x	☐	198-202
**Meta-bias(es)**	16	Specify any planned assessment of meta-bias(es) (e.g., publication bias across studies, selective reporting within studies)	☐	☐	n/a
**Confidence in cumulative evidence**	17	Describe how the strength of the body of evidence will be assessed (e.g., GRADE)	☐	☐	n/a

For peer review only

BMJ Open

The use of metabolomics for predicting spontaneous preterm birth in asymptomatic pregnant women: protocol for a systematic review and meta-analysis

Journal:	*BMJ Open*
Manuscript ID	bmjopen-2018-026033.R2
Article Type:	Protocol
Date Submitted by the Author:	20-Dec-2018
Complete List of Authors:	Souza, Renato; Universidade Estadual de Campinas, Obstetrics and Gynecology<br>Galvão, Rafael; Universidade Estadual de Campinas, Obstetrics and Gynecology<br>Leite, Debora; Campinas' State University, Department of Tocogynecology; Universidade Federal de Pernambuco,<br>Passini Jr, Renato; Universidade Estadual de Campinas Faculdade de Ciencias Medicas<br>Baker, Philip ; University of Leicester, College of Medicine<br>Cecatti, Jose; University of Campinas, Obstetrics and Gynecology
**Primary Subject Heading**:	Obstetrics and gynaecology
Secondary Subject Heading:	Diagnostics
Keywords:	preterm birth, metabolomics, biomarkers, prediction, metabolome

SCHOLARONE™
Manuscripts

The use of metabolomics for predicting spontaneous preterm birth in asymptomatic pregnant women: protocol for a systematic review and meta-analysis

Renato T. Souza [1] (renatotsouzasp@gmail.com)

Rafael Bessa Freitas Galvão [1] (rafaelbfg@gmail.com)

Débora Farias Batista Leite [1,2] (deborafariasleite@gmail.com)

Renato Passini Jr [1] (passini@caism.unicamp.br)

Philip N. Baker [3] (philip.baker@leicester.ac.uk)

José Guilherme Cecatti [1] (cecatti@unicamp.br)

[1] Department of Obstetrics and Gynaecology, University of Campinas, Campinas, São Paulo, Brazil.

[2] Clinics Hospital of Federal University of Pernambuco, Recife, Pernambuco, Brazil.

[3] College of Life Sciences, University of Leicester, England, United Kingdom.

This systematic review protocol is registered at PROSPERO platform (CRD42018100172). Available from: http://www.crd.york.ac.uk/PROSPERO/display_record.php?ID=CRD42018100172.

Corresponding author:

José Guilherme Cecatti

101 Alexander Fleming, Cidade Universitária, campinas, São Paulo, Brazil.

ZIPCODE 13083-881

cecatti@unicamp.br

Word count: 2,233.

For peer review only - http://bmjopen.bmj.com/site/about/guidelines.xhtml

ABSTRACT

Introduction: Preterm birth (PTB) is the leading cause of neonatal mortality and short- and long-term morbidity. The aetiology and pathophysiology of spontaneous PTB are still unclear, which makes the identification of reliable and accurate predictor markers more difficult, particularly for unscreened or asymptomatic women. Metabolomics biomarkers have been demonstrated to be potentially accurate biomarkers for many disorders with complex mechanisms such as PTB. Therefore, we aim to perform a systematic review of metabolomics markers associated with spontaneous PTB. Our research question is "What is the performance of metabolomics for predicting spontaneous preterm birth in asymptomatic pregnant women?"

Methods and analysis: We will focus on studies assessing metabolomics techniques for predicting spontaneous preterm birth in asymptomatic pregnant women. We will conduct a comprehensive systematic review of the literature from the last 10 years. Only observational cohort and case-control studies will be included. Our search strategy will be carried out by two independent reviewers, who will scan title and abstract before carrying out a full review of the article. The scientific databases to be explored include PubMed, MedLine, ScieLo, EMBASE, LILACS, Web of Science, Scopus, and others.

Ethics and dissemination: This systematic review protocol does not require ethical approval. We intend to disseminate our findings in scientific peer-reviewed journal, the Preterm SAMBA study open access website, specialists' conferences, and to our funding agencies.

Registration details: This protocol is registered in PROSPERO platform (code CRD42018100172).

Keywords: preterm birth, spontaneous preterm birth, metabolomics, biomarkers, prediction, metabolome.

For peer review only - http://bmjopen.bmj.com/site/about/guidelines.xhtml

Strengths and limitations of this study

- This systematic review protocol takes into account some important aspects regarding conducting a systematic review about spontaneous preterm birth and metabolomics such as the criteria used for defining spontaneous preterm birth, different population risk stratification, method used to estimate gestational age, and metabolomics techniques details.
- Two independent reviewers are responsible for searching and selecting studies, as also extracting data, and a third reviewer will resolve any disagreement.
- If possible, proper statistical methods will be applied to investigate metabolomics accuracy in predicting spontaneous preterm birth.
- Possible limitations to this review include the different criteria applied for defining spontaneous preterm birth, and the diverse population risk stratification.

For peer review only

INTRODUCTION

Spontaneous preterm birth (sPTB) is the leading cause of perinatal mortality and short- and long-term morbidity [1,2]. It is defined as birth that occurs before 37 weeks gestation due to spontaneous onset of labour or preterm premature rupture of membranes (pPROM) [3,4]. Several pathways and mechanisms linked with preterm birth have been proposed including, neuroendocrine, vascular, immune-inflammatory, and behavioural processes [5]. More specifically, several markers associated with uterine distension/contraction, decidual inflammation/infection and activation of hypothalamic–pituitary–adrenal axis had been studied in the past decades [5,6]. However, no single marker or combination of markers has been found to be accurate enough for predicting sPTB [7–10]. History of previous preterm birth, cervical length at second trimester and cervico-vaginal fetal fibronectin are the most promising clinical tests for predicting spontaneous preterm, but they seem not to be clinically useful for asymptomatic women. Sensitivity of short cervical length (<25mm) and high cervico-vaginal fFN (>50ng/ml) are around 33-36% and 46%, respectively [11–13].

Preterm birth is a complex and multifactorial syndrome that possibly has a long pre-clinical phase, maternal and fetal interactions, genetic and environmental influences, and adaptive mechanisms [14,15]. These challenging aspects, and the presence of still unknown underlying mechanisms, are the main limitations for the identification of an accurate predictor for sPTB [16–18]. None of the predictors used in clinical practice, such as previous history of preterm birth, infection (vaginal and urinary contaminants), fibronectin and transvaginal ultrasonography cervical length demonstrated exceptional accuracy for predicting spontaneous preterm birth [7]. An exploration of innovative approaches is urgently required.

For peer review only - http://bmjopen.bmj.com/site/about/guidelines.xhtml

Metabolomics is the study of metabolites, through identification and quantification of low-weight molecular particles, i.e. tens to hundreds thousands of intermediate products and substrates of systems biology chemical reactions [19,20]. This novel approach has been applied for identifying biomarkers and underlying biochemical pathways associated with complex obstetrical syndromes as preeclampsia, fetal growth restriction, gestational diabetes and preterm birth. In contrast to other "*Omics Sciences*" techniques, metabolomics is more closely associated with the phenotype of the disease and might thus identify a more robust and reliable set of predictors [21]. Importantly, implementing an adequate *Omics* experimental design is crucial for metabolomics studies. Using different baseline population (asymptomatic vs symptomatic or low- vs high-risk women for developing sPTB), study designs (prospective cohorts, case-control or cross sectional studies), sources of samples (amniotic fluid, vaginal fluid, blood, urine, hair, etc) and the timing of sample collection each have significant effects on study findings and the consequent interpretation and contribution to the current gap of knowledge [19].

Different reviews collating scientific knowledge regarding preterm birth biomarkers/predictors has been conducted. Different methodology approaches has been applied so far, including narrative, systematic and umbrella reviews, a more comprehensive review that includes not only original studies but also other reviews [7,19,22,23]. At the best of our knowledge, there is no systematic review on metabolomics markers. Therefore, we aim to conduct a systematic review of original studies investigating the use of metabolomics biomarkers for predicting spontaneous preterm birth in asymptomatic pregnant women. This protocol describes the methods that will be applied in our systematic review.

METHODS AND ANALYSIS

For peer review only

The current systematic review proposal will be conducted, written and published following the Preferred Reporting Items for Systematics Reviews and Meta-Analyses (PRISMA-P) recommendations [24]. Also, it is properly registered at PROSPERO platform – code CRD42018100172.

Review question

What is the performance of metabolomics for predicting spontaneous preterm birth in asymptomatic pregnant women?

Eligibility Criteria

Original cohort or case-control studies involving asymptomatic pregnant women at the moment of sample collection (exposure) and with samples analysed using metabolomics techniques. Studies will be excluded if (1) they are cross-sectional studies, clinical trials, editorials, letter to editors, case reports, expert opinions, commentaries, or any type of review; (2) they describes only experimental studies with animals; or (3) they are duplicated data (e.g. data published in conferences proceedings and, then, published again in scientific journals). In this case, only the most complete publication will be considered, after comparing and confirming that the same technique and metabolites were explored. Studies published from 2008 to 2018 will be considered, and there will be no language restriction. Before submitting this systematic review for publication, we will rerun the search strategy to identify new studies that have been published after performing the first round of search.

Participants

The current review is interested in evaluating the performance of metabolomics biomarkers for spontaneous preterm birth in asymptomatic pregnant women, which may contribute to clinical practice, potentially providing information regarding onset of preterm labour. Nevertheless, we aim to identify studies addressing only early predictors collected from

For peer review only - http://bmjopen.bmj.com/site/about/guidelines.xhtml

women who are in an early preclinical stage, which might contribute to a wider window of opportunity for interventions and also to develop a widely reproducible screening test. Asymptomatic pregnant women should not have regular uterine tightening/contractions or signs of rupture of membranes (i.e. watery discharge). In addition, the study should preferably have a standardized definition of spontaneous preterm birth, the outcome of interest.

Information Sources

The search will be held in the following databases: PubMed, EMBASE, Scopus, CINAHL, and Web of Science, BVS/BIREME, which includes the Latin American and Caribbean Health Sciences Literature (LILACS), Medline and the Scientific Electronic Library Online (Scielo). In addition, secondary sources of original studies will be explored such as Google Scholar, hand-held searching of the reference list of eligible studies, conference proceedings, and contact with authors when necessary.

Search Strategy

The following terms will be used in our search strategy for the different scientific databases: (preterm birth, premature birth, premature infant, premature labor, extremely premature infant, premature obstetric labor, spontaneous preterm birth, extreme preterm birth, late preterm birth, moderate preterm birth, preterm premature rupture of membranes, preterm delivery, PROM, sPTB, preterm PROM, pPROM, p-PROM) AND (metabolomic*, metabonomic*, metabolit*, lipidomic*, H NMR, proton NMR, proton nuclear magnetic resonance, liquid chromatogra*, gas chromatogra*, UPLC, ultra-performance liquid chromatograph*, ultra-performance liquid chromatograph*, HPLC, high performance liquid chrormatograph*, high-performance liquid chrormatograph*) AND (pregnan*, antenat*, ante nat*, prenat*, pre nat*) (Supplementary Material). Respective adaptations in the

For peer review only - http://bmjopen.bmj.com/site/about/guidelines.xhtml

syntax of search for each database will be applied accordingly. No filters - such as "research in animal's models" and "reviews" - will be used in our search strategy, as it will be excluded according to eligibility criteria. The complete search strategy, including Boolean terms, is provided as Supplementary Material.

Data Management

We will export search results to a reference manager (Mendeley®). Then, the following information will be collected from each study using an appropriate form, which will be entered in an Excel® spreadsheet: author's name, year of publication, country, study design, number of participants with and without spontaneous preterm birth, type of metabolomics analysis technique (liquid or gas chromatography, nuclear resonance), laboratory methods for metabolites data acquiring (targeted or untargeted techniques, etc), subtype of preterm birth (spontaneous preterm labour or pPROM), number of fetuses (singleton vs multiple), gestational age when samples were collected, source of samples (type/site of tissue), low or high-risk for preterm birth (authors criteria used to define the population will be collected) and method applied to estimate gestational age. If possible, additional variables related to spontaneous preterm birth categories (delivery before 28 weeks and before 34 weeks) will be recorded for secondary analyses. Original authors will be contacted to clarify data, when needed. Finally, we will check the biochemical class of identified metabolites in Human Metabolome Database (HMDB, version 4.0) to explore and synthetize whether there are common biological pathways associated with spontaneous preterm birth [20].

Selection Process

Two independent reviewers (RTS and RBFG) will be responsible for screening and selecting studies initially according to title or abstract. Both researchers will read the full text of non-excluded studies to discriminate eligibility. A third reviewer (DFBL) will consider any

For peer review only - http://bmjopen.bmj.com/site/about/guidelines.xhtml

disagreement; additional reviewers (RPJ, PNB and JGC) will be responsible for supervising all steps and approving data extraction.

Data Collection Process

We will extract search results to a reference manager where all studies will be stored. Then, included studies will be placed in a new folder. Finally, we will manually extract data of interest from these included studies to an Excel® file. Each reviewer will have their own reference manager account, file and folder and discrepant results will be discussed together with the third reviewer.

Outcomes and Prioritization

The primary outcome is spontaneous preterm birth, defined as any birth occurred before 37 weeks of gestation due to spontaneous onset of labor or preterm premature rupture of membranes. Secondary outcomes are:

1. Spontaneous preterm birth before 28 weeks;
2. Spontaneous preterm birth before 32 weeks;
3. Spontaneous preterm birth before 34 weeks;

The capacity to predict different degrees of sPTB (categories of gestational age) is important as the extreme (<28wks), moderate (<32 weeks) and non-late preterm (<34wks) newborns have different adverse outcomes compared to non-extreme (≥28wks); non-moderate (≥32wks) or late (≥34 wks) preterm newborns.

Ideally, the method of gestational age estimation should be clearly reported. For instance, it can be reported as estimated by last menstrual period (LMP) and confirmed by an early ultrasound or only by an early ultrasound when LMP is unknown/uncertain.

Index test

For peer review only - http://bmjopen.bmj.com/site/about/guidelines.xhtml

Metabolomics techniques to predict spontaneous preterm birth is the diagnostic test of interest. Metabolomics is a technique to identify and quantify metabolites from biological samples using different type of platforms/equipment. The most common platforms include gas, liquid chromatography or ultra-performance liquid chromatography coupled to a mass spectrometer or a proton nuclear magnetic resonance [25]. The performance of the different thresholds of each metabolite will be compared and summarized through hierarchical summary receiver operator characteristic curve (HSROC) (meta-analysis) according to the subgroups described above.

Risk of Bias in individual Studies

We will apply the Quality Assessment of Diagnostic Accuracy Studies (QUADAS-2) tool [26] to assess the risk of bias and applicability of primary diagnostic accuracy studies. Each study will be classified as “low”, “high” or “unclear” regarding risk of bias for each of the four domains of QUADAS tool: Patient Selection, Index Test (metabolomics), Reference Standard (occurrence of preterm birth), and Flow and Timing of participant’s inclusion and follow-up. For example, studies will be labelled as “low” risk of bias for Reference Standard when definition of spontaneous preterm birth and gestational age estimation are clear; “high” risk of bias would be considered when the moment of sample collection is not well described.

Data Synthesis

We will report details of identification, screening, eligibility and included studies using a flow diagram, according to PRISMA recommendations [24]. Data from included studies will be synthetized into tables according to the variables of interest. If possible, we will present data meta-analysis according to study design, metabolomics technique and type of samples analysed. We intend to perform subgroup analysis according to:

- Different metabolomics methods applied: gas or liquid chromatography coupled

For peer review only - http://bmjopen.bmj.com/site/about/guidelines.xhtml

with mass spectrometry or proton nuclear magnetic resonance;

- Singleton and multiple pregnancies;
- Low-risk and high-risk women for developing preterm birth;
- Subtype of preterm birth: Spontaneous preterm birth exclusively due to spontaneous onset of labour with intact membranes or sPTB due to premature rupture of membranes.
- Gestational age interval when samples were collected: 1st trimester, 2nd trimester and 3rd trimester.

Heterogeneity will be assessed by Cochran's Q, Hotelling's T-squared (τ^2) and I^2 tests. Funnel plots and sensitivity and cumulative analyses will be applied for detection of temporal trends and publication bias.

Potential anticipated limitations to this review

Firstly, although we have not considered any language restriction, we consider that there might be a limitation in studies published entirely in non-English language. However, in the last decade, more than 95% of scientific biomedical literature has been published in English [27], then we consider this a minor selection bias. Secondly, we intend to stratify the groups according to population risk. However, the characterization of low- or high-risk for spontaneous preterm birth is controversial and lacks standardization, which might limit data comparison and subgroup analysis. Finally, categorization of sPTB into spontaneous onset of labour or pPROM is another topic of potential limitation - the recognition of the main initial mechanism for preterm delivery might not always be possible. Even when specified, it might provoke uncertainty and could limit further considerations regarding preterm phenotypes. In addition, another limitation is that individual patient data will not be collected.

Patient and Public Involvement

For peer review only

For peer review only - http://bmjopen.bmj.com/site/about/guidelines.xhtml

Patients will not be directly involved in the study and no experience or direct impact from their perspective can be discussed.

ETHICS AND DISSEMINATION

This systematic review does not require ethical approval from the Research Council or Ethics board. We intend to disseminate our findings in scientific peer-reviewed journal, general free access website of Preterm Screening and Metabolomics in Brazil and Auckland (Preterm SAMBA) study, specialists' conferences, and to our funding agencies.

DISCUSSION

This systematic review will comprise current knowledge related with metabolomics in the context of preterm birth prediction. Metabolomics science, a resourceful innovative field that allows better understanding on pathophysiology of complex syndromes, may address the main compounds associated with the spontaneous preterm delivery and, therefore, motivate further researchers to validate early measurable predictors of preterm birth.

Metabolomics performance for predicting sPTB remains unclear and standardized and high-quality studies are needed to clarify the clinical application of metabolites for predicting sPTB. Nevertheless, metabolomics discovery studies commonly requires further validation studies; reproducible methodology is crucial. This systematic review protocol will collate the main potential early biomarkers, subgroup analysis and standardized definition for spontaneous preterm birth to better understand metabolomics performance in predicting sPTB and also to show its heterogeneity in terms of methodology (samples used, metabolomics technique, definition of SPTB phenotype, etc). High performing predictors of preterm birth will help combat this leading cause of neonatal mortality and morbidity.

For peer review only - http://bmjopen.bmj.com/site/about/guidelines.xhtml

For peer review only

For peer review only - http://bmjopen.bmj.com/site/about/guidelines.xhtml

References

1 Lawn JE, Cousens S, Zupan J. 4 million neonatal deaths: when? Where? Why? *Lancet* 2005;**365**:891–900. doi:10.1016/S0140-6736(05)71048-5

2 Howson CP, Kinney M V, McDougall L, *et al.* Born Too Soon: Preterm birth matters. *Reprod Health* 2013;**10 Suppl 1**:S1. doi:10.1186/1742-4755-10-S1-S1

3 Blencowe H, Cousens S, Chou D, *et al.* Born Too Soon: The global epidemiology of 15 million preterm births. *Reprod Health* 2013;**10**:S2. doi:10.1186/1742-4755-10-S1-S2

4 Ananth C V, Vintzileos AM. Epidemiology of preterm birth and its clinical subtypes. *J Matern Fetal Neonatal Med* 2006;**19**:773–82. doi:10.1080/14767050600965882

5 Behrman R, ButlerAS, editors. *Institute of Medicine (IOM). Preterm Birth: Causes, Consequences, and Prevention.* Washington, D.C.: National Academies Press 2007. doi:10.17226/11622

6 Manuck TA, Esplin MS, Biggio J, *et al.* The phenotype of spontaneous preterm birth: application of a clinical phenotyping tool. *Am J Obstet Gynecol* 2015;**212**(4):487.e1-487.e11. doi:10.1016/j.ajog.2015.02.010

7 Honest H, Hyde CJ, Khan KS. Prediction of spontaneous preterm birth: no good test for predicting a spontaneous preterm birth. *Curr Opin Obstet Gynecol* 2012;**24**:422–33. doi:10.1097/GCO.0b013e328359823a

8 Conde-Agudelo A, Papageorghiou A, Kennedy S, *et al.* Novel biomarkers for the prediction of the spontaneous preterm birth phenotype: a systematic review and meta-analysis. *BJOG* 2011;**118**:1042–54. doi:10.1111/j.1471-0528.2011.02923.x

9 Hee L. Likelihood ratios for the prediction of preterm delivery with biomarkers. *Acta Obstet Gynecol Scand* 2011;**90**:1189–99. doi:10.1111/j.1600-0412.2011.01187.x

10 Goldenberg RL, Iams JD, Mercer BM, *et al.* The Preterm Prediction Study: toward a multiple-marker test for spontaneous preterm birth. *Am J Obstet Gynecol* 2001;**185**:643–51. doi:10.1067/mob.2001.116752

11 Iams JD, Goldenberg RL, Meis PJ, *et al.* The length of the cervix and the risk of spontaneous premature delivery. National Institute of Child Health and Human Development Maternal Fetal Medicine Unit Network. *N Engl J Med* 1996;**334**:567–72. doi:10.1056/NEJM199602293340904

12 Smith V, Devane D, Begley CM, *et al.* A systematic review and quality assessment of systematic reviews of fetal fibronectin and transvaginal length for predicting preterm birth. *Eur J Obstet Gynecol Reprod Biol* 2007;**133**:134–42. doi:10.1016/j.ejogrb.2007.03.005

13 Abbott DS, Hezelgrave NL, Seed PT, *et al.* Quantitative fetal fibronectin to predict preterm birth in asymptomatic women at high risk. *Obstet Gynecol* 2015;**125**:1168–

For peer review only

76. doi:10.1097/AOG.0000000000000754

14 Di Renzo GC. The great obstetrical syndromes. *J Matern neonatal Med* 2009;**22**:633–5. doi:10.1080/14767050902866804

15 Brosens I, Pijnenborg R, Vercruysse L, *et al.* The 'Great Obstetrical Syndromes' are associated with disorders of deep placentation. *Am J Obstet Gynecol* 2011;**204**:193–201. doi:10.1016/j.ajog.2010.08.009

16 Practice bulletin no. 130: prediction and prevention of preterm birth. *Obstet Gynecol* 2012;**120**:964–73. doi:10.1097/AOG.0b013e3182723b1b

17 Goldenberg RL, Culhane JF, Iams JD, *et al.* Epidemiology and causes of preterm birth. *Lancet* 2008;**371**:75–84. doi:10.1016/S0140-6736(08)60074-4

18 Honest H, Hyde CJ, Khan KS. Prediction of spontaneous preterm birth. *Curr Opin Obstet Gynecol* 2012;**24**:422–33. doi:10.1097/GCO.0b013e328359823a

19 Horgan RP, Clancy OH, Myers JE, *et al.* An overview of proteomic and metabolomic technologies and their application to pregnancy research. *BJOG* 2009;**116**:173–81. doi:10.1111/j.1471-0528.2008.01997.x

20 Wishart DS, Feunang YD, Marcu A, *et al.* HMDB 4.0: the human metabolome database for 2018. *Nucleic Acids Res* 2018;**46**:D608–17. doi:10.1093/nar/gkx1089

21 Dettmer K, Hammock BD. Metabolomics--a new exciting field within the omics sciences. *Environ Health Perspect* 2004;**112**:A396-7.

22 Lucaroni F, Morciano L, Rizzo G, *et al.* Biomarkers for predicting spontaneous preterm birth: an umbrella systematic review. *J Matern Neonatal Med* 2018;**31**:726–34. doi:10.1080/14767058.2017.1297404

23 Romero R, Espinoza J, Gotsch F, *et al.* The use of high-dimensional biology (genomics, transcriptomics, proteomics, and metabolomics) to understand the preterm parturition syndrome. *BJOG* 2006;**113**:118–35. doi:10.1111/j.1471-0528.2006.01150.x

24 Shamseer L, Moher D, Clarke M, *et al.* Preferred reporting items for systematic review and meta-analysis protocols (PRISMA-P) 2015: elaboration and explanation. *BMJ* 2015;**350**:g7647. doi:10.1136/BMJ.G7647

25 Zhang A, Sun H, Wang P, *et al.* Modern analytical techniques in metabolomics analysis. *Analyst* 2012;**137**:293–300. doi:10.1039/c1an15605e

26 Whiting PF, Rutjes AWS, Westwood ME, *et al.* QUADAS-2: A Revised Tool for the Quality Assessment of Diagnostic Accuracy Studies. *Ann Intern Med* 2011;**155**:529. doi:10.7326/0003-4819-155-8-201110180-00009

27 Rosselli D. The language of biomedical sciences. *Lancet* 2016;**387**:1720–1. doi:10.1016/S0140-6736(16)30259-8

For peer review only - http://bmjopen.bmj.com/site/about/guidelines.xhtml

Author´s Contributions

RTS and RBFG will conduct the systematic review as independent first reviewers. JGC, RPJ and DFBL will decide about conflicting decisions regarding papers selections. PNB, RPJ and JGC participated in the systematic review conception, methodology and framework, together will all the others co-authors.

Funding

This research was supported by Brazilian National Research Council (grant number 401636/2013-5) and Bill and Melinda Gates Foundation (grant number OPP1107597- Grand Challenges Brazil: Reducing the burden of preterm birth, FIOTEC number 05/2013), which provided funding to PRETERM-SAMBA project (www.medscinet.com/samba). RTS and DFL have been awarded PhD scholarships from the CAPES Foundation, an agency under the Ministry of Education of Brazil, process 88881.134095/2016-01 and 8881.134512/2016-01 respectively. The sponsors played no role on the study design or manuscript writing.

Competing interests

All authors are carrying original research about metabolomics and presenting conferences about this topic, including spontaneous preterm birth, preeclampsia, gestational diabetes mellitus and fetal growth restriction. Philip N Baker is principal investigator of Metabolomics Diagnostics Ltd, a company dedicated to develop innovative screening tests using metabolomics technology.

Acknowledgements

For peer review only

For peer review only - http://bmjopen.bmj.com/site/about/guidelines.xhtml

Ana Paula de Morais e Oliveira, librarian of University of Campinas – Unicamp, Brazil, for collaborating in developing search strategy and Rachel Hanisch for her suggestions to some sections of the paper.

Ethics approval and consent to participate

This systematic review does not require ethical approval from the Research Council or Ethics board.

For peer review only

For peer review only - http://bmjopen.bmj.com/site/about/guidelines.xhtml

Seacrh strategy: #1 AND #2 AND #3

1 (OR for each term)	preterm birth<br>premature birth<br>premature infant<br>premature labor<br>extremely premature infant<br>premature obstetric labor<br>spontaneous preterm birth<br>extreme preterm birth<br>late preterm birth<br>moderate preterm birth<br>preterm premature rupture of membranes<br>preterm delivery<br>PROM<br>sPTB<br>preterm PROM<br>pPROM<br>p-PROM
2 (OR for each term)	metabolomic*<br>metabonomic*<br>metabolit*<br>lipidomic*<br>H NMR<br>proton NMR<br>proton nuclear magnetic resonance<br>liquid chromatogra*<br>UPLC<br>ultra-performance liquid chromatograph*<br>ultra performance liquid chromatograph*<br>HPLC<br>high performance liquid chromatograph*<br>high-performance liquid chromatograph*
3 (OR for each term)	pregnan*<br>antenat*<br>ante nat*<br>prenat*<br>pre nat*

For peer review only

PRISMA-P 2015 Checklist

This checklist has been adapted for use with systematic review protocol submissions to BioMed Central journals from Table 3 in Moher D et al**:** Preferred reporting items for systematic review and meta-analysis protocols (PRISMA-P) 2015 statement. *Systematic Reviews* 2015 **4**:1

An Editorial from the Editors-in-Chief of *Systematic Reviews* details why this checklist was adapted - Moher D, Stewart L & Shekelle P**:** Implementing PRISMA-P: recommendations for prospective authors. *Systematic Reviews* 2016 **5**:15

For peer review only

Section/topic	#	Checklist item	Information reported		Line number(s)
			Yes	No	
**ADMINISTRATIVE INFORMATION**					
**Title**					
Identification	1a	Identify the report as a protocol of a systematic review	x		2
Update	1b	If the protocol is for an update of a previous systematic review, identify as such			n/a
**Registration**	2	If registered, provide the name of the registry (e.g., PROSPERO) and registration number in the Abstract	x		48-49
**Authors**					
Contact	3a	Provide name, institutional affiliation, and e-mail address of all protocol authors; provide physical mailing address of corresponding author	x		4-23
Contributions	3b	Describe contributions of protocol authors and identify the guarantor of the review	x		283-287
**Amendments**	4	If the protocol represents an amendment of a previously completed or published protocol, identify as such and list changes; otherwise, state plan for documenting important protocol amendments			n/a
**Support**					
Sources	5a	Indicate sources of financial or other support for the review	x		275-282
Sponsor	5b	Provide name for the review funder and/or sponsor	x		275-282
Role of sponsor/funder	5c	Describe roles of funder(s), sponsor(s), and/or institution(s), if any, in developing the protocol	x		282
**INTRODUCTION**					
**Rationale**	6	Describe the rationale for the review in the context of what is already known	x		51-87
**Objectives**	7	Provide an explicit statement of the question(s) the review will address with reference to	x		94-95

Section/topic	#	Checklist item	Information reported Yes	No	Line number(s)
		participants, interventions, comparators, and outcomes (PICO)			
**METHODS**					
**Eligibility criteria**	8	Specify the study characteristics (e.g., PICO, study design, setting, time frame) and report characteristics (e.g., years considered, language, publication status) to be used as criteria for eligibility for the review	x	☐	96-107
**Information sources**	9	Describe all intended information sources (e.g., electronic databases, contact with study authors, trial registers, or other grey literature sources) with planned dates of coverage	x	☐	119-125
**Search strategy**	10	Present draft of search strategy to be used for at least one electronic database, including planned limits, such that it could be repeated	x	☐	127-136
STUDY RECORDS					
Data management	11a	Describe the mechanism(s) that will be used to manage records and data throughout the review	x	☐	142-155
Selection process	11b	State the process that will be used for selecting studies (e.g., two independent reviewers) through each phase of the review (i.e., screening, eligibility, and inclusion in meta-analysis)	x	☐	157-161
Data collection process	11c	Describe planned method of extracting data from reports (e.g., piloting forms, done independently, in duplicate), any processes for obtaining and confirming data from investigators	x	☐	163-167
**Data items**	12	List and define all variables for which data will be sought (e.g., PICO items, funding sources), any pre-planned data assumptions and simplifications	x	☐	142-155
**Outcomes and prioritization**	13	List and define all outcomes for which data will be sought, including prioritization of main and additional outcomes, with rationale	x	☐	169-179
**Risk of bias in individual studies**	14	Describe anticipated methods for assessing risk of bias of individual studies, including whether this will be done at the outcome or study level, or both; state how this information will be used in data synthesis	x	☐	189-196
DATA					
**Synthesis**	15a	Describe criteria under which study data will be quantitatively synthesized	x	☐	198-209
	15b	If data are appropriate for quantitative synthesis, describe planned summary measures, methods of handling data, and methods of combining data from studies, including any planned exploration of consistency (e.g., I^2, Kendall's tau)	x	☐	185-187
	15c	Describe any proposed additional analyses (e.g., sensitivity or subgroup analyses, meta-regression)	x	☐	185-187

For peer review only

Section/topic	#	Checklist item	Information reported Yes	Information reported No	Line number(s)
	15d	If quantitative synthesis is not appropriate, describe the type of summary planned	x	☐	198-202
**Meta-bias(es)**	16	Specify any planned assessment of meta-bias(es) (e.g., publication bias across studies, selective reporting within studies)	☐	☐	n/a
**Confidence in cumulative evidence**	17	Describe how the strength of the body of evidence will be assessed (e.g., GRADE)	☐	☐	n/a

For peer review only

BMJ Open

The use of metabolomics for predicting spontaneous preterm birth in asymptomatic pregnant women: protocol for a systematic review and meta-analysis

Journal:	*BMJ Open*
Manuscript ID	bmjopen-2018-026033.R3
Article Type:	Protocol
Date Submitted by the Author:	09-Jan-2019
Complete List of Authors:	Souza, Renato; Universidade Estadual de Campinas, Obstetrics and Gynecology<br>Galvão, Rafael; Universidade Estadual de Campinas, Obstetrics and Gynecology<br>Leite, Debora; Campinas' State University, Department of Tocogynecology; Universidade Federal de Pernambuco,<br>Passini Jr, Renato; Universidade Estadual de Campinas Faculdade de Ciencias Medicas<br>Baker, Philip ; University of Leicester, College of Medicine<br>Cecatti, Jose; University of Campinas, Obstetrics and Gynecology
**Primary Subject Heading**:	Obstetrics and gynaecology
Secondary Subject Heading:	Diagnostics
Keywords:	preterm birth, metabolomics, biomarkers, prediction, metabolome

SCHOLARONE™
Manuscripts

The use of metabolomics for predicting spontaneous preterm birth in asymptomatic pregnant women: protocol for a systematic review and meta-analysis

Renato T. Souza [1] (renatotsouzasp@gmail.com)
Rafael Bessa Freitas Galvão [1] (rafaelbfg@gmail.com)
Débora Farias Batista Leite [1,2] (deborafariasleite@gmail.com)
Renato Passini Jr [1] (passini@caism.unicamp.br)
Philip N. Baker [3] (philip.baker@leicester.ac.uk)
José Guilherme Cecatti [1] (cecatti@unicamp.br)

[1] Department of Obstetrics and Gynaecology, University of Campinas, Campinas, São Paulo, Brazil.
[2] Clinics Hospital of Federal University of Pernambuco, Recife, Pernambuco, Brazil.
[3] College of Life Sciences, University of Leicester, England, United Kingdom.

This systematic review protocol is registered at PROSPERO platform (CRD42018100172). Available from: http://www.crd.york.ac.uk/PROSPERO/display_record.php?ID=CRD42018100172.

Corresponding author:
José Guilherme Cecatti
101 Alexander Fleming, Cidade Universitária, campinas, São Paulo, Brazil.
ZIPCODE 13083-881
cecatti@unicamp.br

Word count: 2,233.

For peer review only

ABSTRACT

Introduction: Preterm birth (PTB) is the leading cause of neonatal mortality and short- and long-term morbidity. The aetiology and pathophysiology of spontaneous PTB are still unclear, which makes the identification of reliable and accurate predictor markers more difficult, particularly for unscreened or asymptomatic women. Metabolomics biomarkers have been demonstrated to be potentially accurate biomarkers for many disorders with complex mechanisms such as PTB. Therefore, we aim to perform a systematic review of metabolomics markers associated with spontaneous PTB. Our research question is "What is the performance of metabolomics for predicting spontaneous preterm birth in asymptomatic pregnant women?"

Methods and analysis: We will focus on studies assessing metabolomics techniques for predicting spontaneous preterm birth in asymptomatic pregnant women. We will conduct a comprehensive systematic review of the literature from the last 10 years. Only observational cohort and case-control studies will be included. Our search strategy will be carried out by two independent reviewers, who will scan title and abstract before carrying out a full review of the article. The scientific databases to be explored include PubMed, MedLine, ScieLo, EMBASE, LILACS, Web of Science, Scopus, and others.

Ethics and dissemination: This systematic review protocol does not require ethical approval. We intend to disseminate our findings in scientific peer-reviewed journal, the Preterm SAMBA study open access website, specialists' conferences, and to our funding agencies.

Registration details: This protocol is registered in PROSPERO platform (code CRD42018100172).

Keywords: preterm birth, spontaneous preterm birth, metabolomics, biomarkers, prediction, metabolome.

For peer review only

For peer review only - http://bmjopen.bmj.com/site/about/guidelines.xhtml

Strengths and limitations of this study

- This systematic review protocol takes into account some important aspects regarding conducting a systematic review about spontaneous preterm birth and metabolomics such as the criteria used for defining spontaneous preterm birth, different population risk stratification, method used to estimate gestational age, and metabolomics techniques details.
- Two independent reviewers are responsible for searching and selecting studies, as also extracting data, and a third reviewer will resolve any disagreement.
- If possible, proper statistical methods will be applied to investigate metabolomics accuracy in predicting spontaneous preterm birth.
- Possible limitations to this review include the different criteria applied for defining spontaneous preterm birth, and the diverse population risk stratification.

For peer review only

For peer review only - http://bmjopen.bmj.com/site/about/guidelines.xhtml

INTRODUCTION

Spontaneous preterm birth (sPTB) is the leading cause of perinatal mortality and short- and long-term morbidity [1,2]. It is defined as birth that occurs before 37 weeks gestation due to spontaneous onset of labour or preterm premature rupture of membranes (pPROM) [3,4]. Several pathways and mechanisms linked with preterm birth have been proposed including, neuroendocrine, vascular, immune-inflammatory, and behavioural processes [5]. More specifically, several markers associated with uterine distension/contraction, decidual inflammation/infection and activation of hypothalamic–pituitary–adrenal axis had been studied in the past decades [5,6]. However, no single marker or combination of markers has been found to be accurate enough for predicting sPTB [7–10]. History of previous preterm birth, cervical length at second trimester and cervico-vaginal fetal fibronectin are the most promising clinical tests for predicting spontaneous preterm, but they seem not to be clinically useful for asymptomatic women. Sensitivity of short cervical length (<25mm) and high cervico-vaginal fFN (>50ng/ml) are around 33-36% and 46%, respectively [11–13].

Preterm birth is a complex and multifactorial syndrome that possibly has a long pre-clinical phase, maternal and fetal interactions, genetic and environmental influences, and adaptive mechanisms [14,15]. These challenging aspects, and the presence of still unknown underlying mechanisms, are the main limitations for the identification of an accurate predictor for sPTB [16–18]. None of the predictors used in clinical practice, such as previous history of preterm birth, infection (vaginal and urinary contaminants), fibronectin and transvaginal ultrasonography cervical length demonstrated exceptional accuracy for predicting spontaneous preterm birth [7]. An exploration of innovative approaches is urgently required.

For peer review only - http://bmjopen.bmj.com/site/about/guidelines.xhtml

Metabolomics is the study of metabolites, through identification and quantification of low-weight molecular particles, i.e. tens to hundreds thousands of intermediate products and substrates of systems biology chemical reactions [19,20]. This novel approach has been applied for identifying biomarkers and underlying biochemical pathways associated with complex obstetrical syndromes as preeclampsia, fetal growth restriction, gestational diabetes and preterm birth. In contrast to other "*Omics Sciences*" techniques, metabolomics is more closely associated with the phenotype of the disease and might thus identify a more robust and reliable set of predictors [21]. Importantly, implementing an adequate *Omics* experimental design is crucial for metabolomics studies. Using different baseline population (asymptomatic vs symptomatic or low- vs high-risk women for developing sPTB), study designs (prospective cohorts, case-control or cross sectional studies), sources of samples (amniotic fluid, vaginal fluid, blood, urine, hair, etc) and the timing of sample collection each have significant effects on study findings and the consequent interpretation and contribution to the current gap of knowledge [19].

Different reviews collating scientific knowledge regarding preterm birth biomarkers/predictors has been conducted. Different methodology approaches has been applied so far, including narrative, systematic and umbrella reviews, a more comprehensive review that includes not only original studies but also other reviews [7,22–24]. At the best of our knowledge, there is no systematic review on metabolomics markers. Therefore, we aim to conduct a systematic review of original studies investigating the use of metabolomics biomarkers for predicting spontaneous preterm birth in asymptomatic pregnant women. This protocol describes the methods that will be applied in our systematic review.

METHODS AND ANALYSIS

For peer review only - http://bmjopen.bmj.com/site/about/guidelines.xhtml

The current systematic review proposal will be conducted, written and published following the Preferred Reporting Items for Systematics Reviews and Meta-Analyses (PRISMA-P) recommendations [25]. Also, it is properly registered at PROSPERO platform – code CRD42018100172.

Review question

What is the performance of metabolomics for predicting spontaneous preterm birth in asymptomatic pregnant women?

Eligibility Criteria

Original cohort or case-control studies involving asymptomatic pregnant women at the moment of sample collection (exposure) and with samples analysed using metabolomics techniques. Studies will be excluded if (1) they are cross-sectional studies, clinical trials, editorials, letter to editors, case reports, expert opinions, commentaries, or any type of review; (2) they describes only experimental studies with animals; or (3) they are duplicated data (e.g. data published in conferences proceedings and, then, published again in scientific journals). In this case, only the most complete publication will be considered, after comparing and confirming that the same technique and metabolites were explored. Studies published from 2008 to 2018 will be considered, and there will be no language restriction. Before submitting this systematic review for publication, we will rerun the search strategy to identify new studies that have been published after performing the first round of search.

Participants

The current review is interested in evaluating the performance of metabolomics biomarkers for spontaneous preterm birth in asymptomatic pregnant women, which may contribute to clinical practice, potentially providing information regarding onset of preterm labour. Nevertheless, we aim to identify studies addressing only early predictors collected from

For peer review only - http://bmjopen.bmj.com/site/about/guidelines.xhtml

women who are in an early preclinical stage, which might contribute to a wider window of opportunity for interventions and also to develop a widely reproducible screening test. Asymptomatic pregnant women should not have regular uterine tightening/contractions or signs of rupture of membranes (i.e. watery discharge). In addition, the study should preferably have a standardized definition of spontaneous preterm birth, the outcome of interest.

Information Sources

The search will be held in the following databases: PubMed, EMBASE, Scopus, CINAHL, and Web of Science, BVS/BIREME, which includes the Latin American and Caribbean Health Sciences Literature (LILACS), Medline and the Scientific Electronic Library Online (Scielo). In addition, secondary sources of original studies will be explored such as Google Scholar, hand-held searching of the reference list of eligible studies, conference proceedings, and contact with authors when necessary.

Search Strategy

The following terms will be used in our search strategy for the different scientific databases: (preterm birth, premature birth, premature infant, premature labor, extremely premature infant, premature obstetric labor, spontaneous preterm birth, extreme preterm birth, late preterm birth, moderate preterm birth, preterm premature rupture of membranes, preterm delivery, PROM, sPTB, preterm PROM, pPROM, p-PROM) AND (metabolomic*, metabonomic*, metabolit*, lipidomic*, H NMR, proton NMR, proton nuclear magnetic resonance, liquid chromatogra*, gas chromatogra*, UPLC, ultra-performance liquid chromatograph*, ultra-performance liquid chromatograph*, HPLC, high performance liquid chrormatograph*, high-performance liquid chrormatograph*) AND (pregnan*, antenat*, ante nat*, prenat*, pre nat*) (Supplementary Material). Respective adaptations in the

For peer review only - http://bmjopen.bmj.com/site/about/guidelines.xhtml

syntax of search for each database will be applied accordingly. No filters - such as “research in animal’s models” and “reviews” - will be used in our search strategy, as it will be excluded according to eligibility criteria. The complete search strategy, including Boolean terms, is provided as Supplementary Material.

Data Management

We will export search results to a reference manager (Mendeley®). Then, the following information will be collected from each study using an appropriate form, which will be entered in an Excel® spreadsheet: author’s name, year of publication, country, study design, number of participants with and without spontaneous preterm birth, type of metabolomics analysis technique (liquid or gas chromatography, nuclear resonance), laboratory methods for metabolites data acquiring (targeted or untargeted techniques, etc), subtype of preterm birth (spontaneous preterm labour or pPROM), number of fetuses (singleton vs multiple), gestational age when samples were collected, source of samples (type/site of tissue), low or high-risk for preterm birth (authors criteria used to define the population will be collected) and method applied to estimate gestational age. If possible, additional variables related to spontaneous preterm birth categories (delivery before 28 weeks and before 34 weeks) will be recorded for secondary analyses. Original authors will be contacted to clarify data, when needed. Finally, we will check the biochemical class of identified metabolites in Human Metabolome Database (HMDB, version 4.0) to explore and synthetize whether there are common biological pathways associated with spontaneous preterm birth [20].

Selection Process

Two independent reviewers (RTS and RBFG) will be responsible for screening and selecting studies initially according to title or abstract. Both researchers will read the full text of non-excluded studies to discriminate eligibility. A third reviewer (DFBL) will consider any

For peer review only - http://bmjopen.bmj.com/site/about/guidelines.xhtml

disagreement; additional reviewers (RPJ, PNB and JGC) will be responsible for supervising all steps and approving data extraction.

Data Collection Process

We will extract search results to a reference manager where all studies will be stored. Then, included studies will be placed in a new folder. Finally, we will manually extract data of interest from these included studies to an Excel® file. Each reviewer will have their own reference manager account, file and folder and discrepant results will be discussed together with the third reviewer.

Outcomes and Prioritization

The primary outcome is spontaneous preterm birth, defined as any birth occurred before 37 weeks of gestation due to spontaneous onset of labor or preterm premature rupture of membranes. Secondary outcomes are:

1. Spontaneous preterm birth before 28 weeks;
2. Spontaneous preterm birth before 32 weeks;
3. Spontaneous preterm birth before 34 weeks;

The capacity to predict different degrees of sPTB (categories of gestational age) is important as the extreme (<28wks), moderate (<32 weeks) and non-late preterm (<34wks) newborns have different adverse outcomes compared to non-extreme (≥28wks); non-moderate (≥32wks) or late (≥34 wks) preterm newborns.

Ideally, the method of gestational age estimation should be clearly reported. For instance, it can be reported as estimated by last menstrual period (LMP) and confirmed by an early ultrasound or only by an early ultrasound when LMP is unknown/uncertain.

Index test

For peer review only - http://bmjopen.bmj.com/site/about/guidelines.xhtml

Metabolomics techniques to predict spontaneous preterm birth is the diagnostic test of interest. Metabolomics is a technique to identify and quantify metabolites from biological samples using different type of platforms/equipment. The most common platforms include gas, liquid chromatography or ultra-performance liquid chromatography coupled to a mass spectrometer or a proton nuclear magnetic resonance [26]. The performance of the different thresholds of each metabolite will be compared and summarized through hierarchical summary receiver operator characteristic curve (HSROC) (meta-analysis) according to the subgroups described above. Considering that the raw data is not available in the majority of the diagnostic test accuracy studies [27] and that metabolites levels are usually reported as continuous variables, we intend to use a meta-analysis model based on ROC curves [28]. Briefly, a two-parameter model, based on the estimation of α and β parameters (using standard errors or maximum likelihood), will be applied as reported by Kester & Buntinx [28]. Therefore, pooled ROC curves can be converted to a estimated ROC curve with 95% confidence interval. This method can also be applied in categorical-ordinal variables tests.

Risk of Bias in individual Studies

We will apply the Quality Assessment of Diagnostic Accuracy Studies (QUADAS-2) tool [29] to assess the risk of bias and applicability of primary diagnostic accuracy studies. Each study will be classified as “low”, “high” or “unclear” regarding risk of bias for each of the four domains of QUADAS tool: Patient Selection, Index Test (metabolomics), Reference Standard (occurrence of preterm birth), and Flow and Timing of participant’s inclusion and follow-up. For example, studies will be labelled as “low” risk of bias for Reference Standard when definition of spontaneous preterm birth and gestational age estimation are clear; “high” risk of bias would be considered when the moment of sample collection is not well described.

For peer review only - http://bmjopen.bmj.com/site/about/guidelines.xhtml

Data Synthesis

We will report details of identification, screening, eligibility and included studies using a flow diagram, according to PRISMA recommendations [25]. Data from included studies will be synthetized into tables according to the variables of interest. If possible, we will present data meta-analysis according to study design, metabolomics technique and type of samples analysed. We intend to perform subgroup analysis according to:

- Different metabolomics methods applied: gas or liquid chromatography coupled with mass spectrometry or proton nuclear magnetic resonance;
- Singleton and multiple pregnancies;
- Low-risk and high-risk women for developing preterm birth;
- Subtype of preterm birth: Spontaneous preterm birth exclusively due to spontaneous onset of labour with intact membranes or sPTB due to premature rupture of membranes.
- Gestational age interval when samples were collected: 1st trimester, 2nd trimester and 3rd trimester.

Heterogeneity will be assessed by Cochran's Q, Hotelling's T-squared (τ^2) and I^2 tests. Funnel plots and sensitivity and cumulative analyses will be applied for detection of temporal trends and publication bias.

Potential anticipated limitations to this review

Firstly, although we have not considered any language restriction, we consider that there might be a limitation in studies published entirely in non-English language. However, in the last decade, more than 95% of scientific biomedical literature has been published in English [30], then we consider this a minor selection bias. Secondly, we intend to stratify the groups according to population risk. However, the characterization of low- or high-risk for

For peer review only - http://bmjopen.bmj.com/site/about/guidelines.xhtml

spontaneous preterm birth is controversial and lacks standardization, which might limit data comparison and subgroup analysis. Finally, categorization of sPTB into spontaneous onset of labour or pPROM is another topic of potential limitation - the recognition of the main initial mechanism for preterm delivery might not always be possible. Even when specified, it might provoke uncertainty and could limit further considerations regarding preterm phenotypes. In addition, another limitation is that individual patient data will not be collected.

Patient and Public Involvement

Patients will not be directly involved in the study and no experience or direct impact from their perspective can be discussed.

ETHICS AND DISSEMINATION

This systematic review does not require ethical approval from the Research Council or Ethics board. We intend to disseminate our findings in scientific peer-reviewed journal, general free access website of Preterm Screening and Metabolomics in Brazil and Auckland (Preterm SAMBA) study, specialists' conferences, and to our funding agencies.

DISCUSSION

This systematic review will comprise current knowledge related with metabolomics in the context of preterm birth prediction. Metabolomics science, a resourceful innovative field that allows better understanding on pathophysiology of complex syndromes, may address the main compounds associated with the spontaneous preterm delivery and, therefore, motivate further researchers to validate early measurable predictors of preterm birth.

Metabolomics performance for predicting sPTB remains unclear and standardized and high-quality studies are needed to clarify the clinical application of metabolites for predicting

For peer review only

For peer review only - http://bmjopen.bmj.com/site/about/guidelines.xhtml

sPTB. Nevertheless, metabolomics discovery studies commonly requires further validation studies; reproducible methodology is crucial. This systematic review protocol will collate the main potential early biomarkers, subgroup analysis and standardized definition for spontaneous preterm birth to better understand metabolomics performance in predicting sPTB and also to show its heterogeneity in terms of methodology (samples used, metabolomics technique, definition of SPTB phenotype, etc). High performing predictors of preterm birth will help combat this leading cause of neonatal mortality and morbidity.

For peer review only

For peer review only - http://bmjopen.bmj.com/site/about/guidelines.xhtml

References

1 Lawn JE, Cousens S, Zupan J. 4 million neonatal deaths: when? Where? Why? *Lancet* 2005;**365**:891–900. doi:10.1016/S0140-6736(05)71048-5

2 Howson CP, Kinney M V, McDougall L, *et al.* Born Too Soon: Preterm birth matters. *Reprod Health* 2013;**10 Suppl 1**:S1. doi:10.1186/1742-4755-10-S1-S1

3 Blencowe H, Cousens S, Chou D, *et al.* Born Too Soon: The global epidemiology of 15 million preterm births. *Reprod Health* 2013;**10**:S2. doi:10.1186/1742-4755-10-S1-S2

4 Ananth C V, Vintzileos AM. Epidemiology of preterm birth and its clinical subtypes. *J Matern Fetal Neonatal Med* 2006;**19**:773–82. doi:10.1080/14767050600965882

5 Behrman R, ButlerAS, editors. *Institute of Medicine (IOM). Preterm Birth: Causes, Consequences, and Prevention.* Washington, D.C.: : National Academies Press 2007. doi:10.17226/11622

6 Manuck TA, Esplin MS, Biggio J, *et al.* The phenotype of spontaneous preterm birth: application of a clinical phenotyping tool. *Am J Obstet Gynecol* Published Online First: February 2015. doi:10.1016/j.ajog.2015.02.010

7 Honest H, Hyde CJ, Khan KS. Prediction of spontaneous preterm birth: no good test for predicting a spontaneous preterm birth. *Curr Opin Obstet Gynecol* 2012;**24**:422–33. doi:10.1097/GCO.0b013e328359823a

8 Conde-Agudelo A, Papageorghiou A, Kennedy S, *et al.* Novel biomarkers for the prediction of the spontaneous preterm birth phenotype: a systematic review and meta-analysis. *BJOG An Int J Obstet Gynaecol* 2011;**118**:1042–54. doi:10.1111/j.1471-0528.2011.02923.x

9 Hee L. Likelihood ratios for the prediction of preterm delivery with biomarkers. *Acta Obstet Gynecol Scand* 2011;**90**:1189–99. doi:10.1111/j.1600-0412.2011.01187.x

10 Goldenberg RL, Iams JD, Mercer BM, *et al.* The Preterm Prediction Study: toward a multiple-marker test for spontaneous preterm birth. *Am J Obstet Gynecol* 2001;**185**:643–51. doi:10.1067/mob.2001.116752

11 Iams JD, Goldenberg RL, Meis PJ, *et al.* The length of the cervix and the risk of spontaneous premature delivery. National Institute of Child Health and Human Development Maternal Fetal Medicine Unit Network. *N Engl J Med* 1996;**334**:567–72. doi:10.1056/NEJM199602293340904

12 Smith V, Devane D, Begley CM, *et al.* A systematic review and quality assessment of systematic reviews of fetal fibronectin and transvaginal length for predicting preterm birth. *Eur J Obstet Gynecol Reprod Biol* 2007;**133**:134–42. doi:10.1016/j.ejogrb.2007.03.005

13 Abbott DS, Hezelgrave NL, Seed PT, *et al.* Quantitative fetal fibronectin to predict

For peer review only - http://bmjopen.bmj.com/site/about/guidelines.xhtml

preterm birth in asymptomatic women at high risk. *Obstet Gynecol* 2015;**125**:1168–76. doi:10.1097/AOG.0000000000000754

14 Di Renzo GC. The great obstetrical syndromes. *J Matern neonatal Med* 2009;**22**:633–5. doi:10.1080/14767050902866804

15 Brosens I, Pijnenborg R, Vercruysse L, *et al.* The 'Great Obstetrical Syndromes' are associated with disorders of deep placentation. *Am J Obstet Gynecol* 2011;**204**:193–201. doi:10.1016/j.ajog.2010.08.009

16 Practice bulletin no. 130: prediction and prevention of preterm birth. *Obstet Gynecol* 2012;**120**:964–73. doi:10.1097/AOG.0b013e3182723b1b

17 Goldenberg RL, Culhane JF, Iams JD, *et al.* Epidemiology and causes of preterm birth. *Lancet* 2008;**371**:75–84. doi:10.1016/S0140-6736(08)60074-4

18 Honest H, Hyde CJ, Khan KS. Prediction of spontaneous preterm birth. *Curr Opin Obstet Gynecol* 2012;**24**:422–33. doi:10.1097/GCO.0b013e328359823a

19 Horgan RP, Clancy OH, Myers JE, *et al.* An overview of proteomic and metabolomic technologies and their application to pregnancy research. *BJOG* 2009;**116**:173–81. doi:10.1111/j.1471-0528.2008.01997.x

20 Wishart DS, Feunang YD, Marcu A, *et al.* HMDB 4.0: the human metabolome database for 2018. *Nucleic Acids Res* 2018;**46**:D608–17. doi:10.1093/nar/gkx1089

21 Dettmer K, Hammock BD. Metabolomics--a new exciting field within the omics sciences. *Environ Health Perspect* 2004;**112**:A396-7.http://www.ncbi.nlm.nih.gov/pubmed/15159211 (accessed 5 Sep 2017).

22 Lucaroni F, Morciano L, Rizzo G, *et al.* Biomarkers for predicting spontaneous preterm birth: an umbrella systematic review. *J Matern Neonatal Med* 2018;**31**:726–34. doi:10.1080/14767058.2017.1297404

23 Horgan RP, Clancy OH, Myers JE, *et al.* An overview of proteomic and metabolomic technologies and their application to pregnancy research. *BJOG* 2009;**116**:173–81. doi:10.1111/j.1471-0528.2008.01997.x

24 Romero R, Espinoza J, Gotsch F, *et al.* The use of high-dimensional biology (genomics, transcriptomics, proteomics, and metabolomics) to understand the preterm parturition syndrome. *BJOG An Int J Obstet Gynaecol* 2006;**113**:118–35. doi:10.1111/j.1471-0528.2006.01150.x

25 Shamseer L, Moher D, Clarke M, *et al.* Preferred reporting items for systematic review and meta-analysis protocols (PRISMA-P) 2015: elaboration and explanation. *BMJ* 2015;**350**:g7647. doi:10.1136/BMJ.G7647

26 Zhang A, Sun H, Wang P, *et al.* Modern analytical techniques in metabolomics analysis. *Analyst* 2012;**137**:293–300. doi:10.1039/c1an15605e

For peer review only

For peer review only - http://bmjopen.bmj.com/site/about/guidelines.xhtml

27 McGrath TA, Alabousi M, Skidmore B, *et al.* Recommendations for reporting of systematic reviews and meta-analyses of diagnostic test accuracy: a systematic review. *Syst Rev* 2017;**6**:194. doi:10.1186/s13643-017-0590-8

28 Kester ADM, Buntinx F. Meta-analysis of ROC Curves. *Med Decis Mak* 2000;**20**:430–9. doi:10.1177/0272989X0002000407

29 Whiting PF, Rutjes AWS, Westwood ME, *et al.* QUADAS-2: A Revised Tool for the Quality Assessment of Diagnostic Accuracy Studies. *Ann Intern Med* 2011;**155**:529. doi:10.7326/0003-4819-155-8-201110180-00009

30 Rosselli D. The language of biomedical sciences. *Lancet* 2016;**387**:1720–1. doi:10.1016/S0140-6736(16)30259-8

Author´s Contributions

RTS and RBFG will conduct the systematic review as independent first reviewers. JGC, RPJ and DFBL will decide about conflicting decisions regarding papers selections. PNB, RPJ and JGC participated in the systematic review conception, methodology and framework, together will all the others co-authors.

Funding

This research was supported by Brazilian National Research Council (grant number 401636/2013-5) and Bill and Melinda Gates Foundation (grant number OPP1107597- Grand Challenges Brazil: Reducing the burden of preterm birth, FIOTEC number 05/2013), which provided funding to PRETERM-SAMBA project (www.medscinet.com/samba). RTS and DFL have been awarded PhD scholarships from the CAPES Foundation, an agency under the Ministry of Education of Brazil, process 88881.134095/2016-01 and 8881.134512/2016-01 respectively. The sponsors played no role on the study design or manuscript writing.

Competing interests

For peer review only

All authors are carrying original research about metabolomics and presenting conferences about this topic, including spontaneous preterm birth, preeclampsia, gestational diabetes mellitus and fetal growth restriction. Philip N Baker is principal investigator of Metabolomics Diagnostics Ltd, a company dedicated to develop innovative screening tests using metabolomics technology.

Acknowledgements

Ana Paula de Morais e Oliveira, librarian of University of Campinas – Unicamp, Brazil, for collaborating in developing search strategy and Rachel Hanisch for her suggestions to some sections of the paper.

Ethics approval and consent to participate

This systematic review does not require ethical approval from the Research Council or Ethics board.

For peer review only

For peer review only - http://bmjopen.bmj.com/site/about/guidelines.xhtml

Seacrh strategy: #1 AND #2 AND #3

1 (OR for each term)	preterm birth<br>premature birth<br>premature infant<br>premature labor<br>extremely premature infant<br>premature obstetric labor<br>spontaneous preterm birth<br>extreme preterm birth<br>late preterm birth<br>moderate preterm birth<br>preterm premature rupture of membranes<br>preterm delivery<br>PROM<br>sPTB<br>preterm PROM<br>pPROM<br>p-PROM
2 (OR for each term)	metabolomic*<br>metabonomic*<br>metabolit*<br>lipidomic*<br>H NMR<br>proton NMR<br>proton nuclear magnetic resonance<br>liquid chromatogra*<br>UPLC<br>ultra-performance liquid chromatograph*<br>ultra performance liquid chromatograph*<br>HPLC<br>high performance liquid chromatograph*<br>high-performance liquid chromatograph*
3 (OR for each term)	pregnan*<br>antenat*<br>ante nat*<br>prenat*<br>pre nat*

For peer review only

PRISMA-P 2015 Checklist

This checklist has been adapted for use with systematic review protocol submissions to BioMed Central journals from Table 3 in Moher D et al**:** Preferred reporting items for systematic review and meta-analysis protocols (PRISMA-P) 2015 statement. *Systematic Reviews* 2015 **4**:1

An Editorial from the Editors-in-Chief of *Systematic Reviews* details why this checklist was adapted - Moher D, Stewart L & Shekelle P**:** Implementing PRISMA-P: recommendations for prospective authors. *Systematic Reviews* 2016 **5**:15

Section/topic	#	Checklist item	Information reported Yes	Information reported No	Line number(s)
**ADMINISTRATIVE INFORMATION**					
**Title**					
Identification	1a	Identify the report as a protocol of a systematic review	x	☐	2
Update	1b	If the protocol is for an update of a previous systematic review, identify as such	☐	☐	n/a
**Registration**	2	If registered, provide the name of the registry (e.g., PROSPERO) and registration number in the Abstract	x	☐	48-49
**Authors**					
Contact	3a	Provide name, institutional affiliation, and e-mail address of all protocol authors; provide physical mailing address of corresponding author	x	☐	4-23
Contributions	3b	Describe contributions of protocol authors and identify the guarantor of the review	x	☐	283-287
**Amendments**	4	If the protocol represents an amendment of a previously completed or published protocol, identify as such and list changes; otherwise, state plan for documenting important protocol amendments	☐	☐	n/a
**Support**					
Sources	5a	Indicate sources of financial or other support for the review	x	☐	275-282
Sponsor	5b	Provide name for the review funder and/or sponsor	x	☐	275-282
Role of sponsor/funder	5c	Describe roles of funder(s), sponsor(s), and/or institution(s), if any, in developing the protocol	x	☐	282
**INTRODUCTION**					
**Rationale**	6	Describe the rationale for the review in the context of what is already known	x	☐	51-87
**Objectives**	7	Provide an explicit statement of the question(s) the review will address with reference to	x	☐	94-95

For peer review only

Section/topic	#	Checklist item	Information reported		Line number(s)
			Yes	No	
		participants, interventions, comparators, and outcomes (PICO)			
**METHODS**					
**Eligibility criteria**	8	Specify the study characteristics (e.g., PICO, study design, setting, time frame) and report characteristics (e.g., years considered, language, publication status) to be used as criteria for eligibility for the review	x	☐	96-107
**Information sources**	9	Describe all intended information sources (e.g., electronic databases, contact with study authors, trial registers, or other grey literature sources) with planned dates of coverage	x	☐	119-125
**Search strategy**	10	Present draft of search strategy to be used for at least one electronic database, including planned limits, such that it could be repeated	x	☐	127-136
STUDY RECORDS					
Data management	11a	Describe the mechanism(s) that will be used to manage records and data throughout the review	x	☐	142-155
Selection process	11b	State the process that will be used for selecting studies (e.g., two independent reviewers) through each phase of the review (i.e., screening, eligibility, and inclusion in meta-analysis)	x	☐	157-161
Data collection process	11c	Describe planned method of extracting data from reports (e.g., piloting forms, done independently, in duplicate), any processes for obtaining and confirming data from investigators	x	☐	163-167
**Data items**	12	List and define all variables for which data will be sought (e.g., PICO items, funding sources), any pre-planned data assumptions and simplifications	x	☐	142-155
**Outcomes and prioritization**	13	List and define all outcomes for which data will be sought, including prioritization of main and additional outcomes, with rationale	x	☐	169-179
**Risk of bias in individual studies**	14	Describe anticipated methods for assessing risk of bias of individual studies, including whether this will be done at the outcome or study level, or both; state how this information will be used in data synthesis	x	☐	189-196
DATA					
**Synthesis**	15a	Describe criteria under which study data will be quantitatively synthesized	x	☐	198-209
	15b	If data are appropriate for quantitative synthesis, describe planned summary measures, methods of handling data, and methods of combining data from studies, including any planned exploration of consistency (e.g., I^2, Kendall's tau)	x	☐	185-187
	15c	Describe any proposed additional analyses (e.g., sensitivity or subgroup analyses, meta-regression)	x	☐	185-187

For peer review only

Section/topic	#	Checklist item	Information reported Yes	Information reported No	Line number(s)
	15d	If quantitative synthesis is not appropriate, describe the type of summary planned	x	☐	198-202
**Meta-bias(es)**	16	Specify any planned assessment of meta-bias(es) (e.g., publication bias across studies, selective reporting within studies)	☐	☐	n/a
**Confidence in cumulative evidence**	17	Describe how the strength of the body of evidence will be assessed (e.g., GRADE)	☐	☐	n/a

For peer review only
